# Efficient recombinant production of prodigiosin in *Pseudomonas putida*

**DOI:** 10.3389/fmicb.2015.00972

**Published:** 2015-09-15

**Authors:** Andreas Domröse, Andreas S. Klein, Jennifer Hage-Hülsmann, Stephan Thies, Vera Svensson, Thomas Classen, Jörg Pietruszka, Karl-Erich Jaeger, Thomas Drepper, Anita Loeschcke

**Affiliations:** ^1^Institute of Molecular Enzyme Technology, Heinrich Heine University Düsseldorf, Forschungszentrum Jülich GmbHJülich, Germany; ^2^Institute of Bioorganic Chemistry, Heinrich Heine University Düsseldorf, Forschungszentrum Jülich GmbHJülich, Germany; ^3^Institute of Bio- and Geosciences (IBG-1): Biotechnology, Forschungszentrum Jülich GmbHJülich, Germany

**Keywords:** prodigiosin, *Pseudomonas putida*, heterologous production, extraction, purification

## Abstract

*Serratia marcescens* and several other bacteria produce the red-colored pigment prodigiosin which possesses bioactivities as an antimicrobial, anticancer, and immunosuppressive agent. Therefore, there is a great interest to produce this natural compound. Efforts aiming at its biotechnological production have so far largely focused on the original producer and opportunistic human pathogen *S. marcescens*. Here, we demonstrate efficient prodigiosin production in the heterologous host *Pseudomonas putida*. Random chromosomal integration of the 21 kb prodigiosin biosynthesis gene cluster of *S. marcescens* in *P. putida* KT2440 was employed to construct constitutive prodigiosin production strains. Standard cultivation parameters were optimized such that titers of 94 mg/L culture were obtained upon growth of *P. putida* at 20°C using rich medium under high aeration conditions. Subsequently, a novel, fast and effective protocol for prodigiosin extraction and purification was established enabling the straightforward isolation of prodigiosin from *P. putida* growth medium. In summary, we describe here a highly efficient method for the heterologous biosynthetic production of prodigiosin which may serve as a basis to produce large amounts of this bioactive natural compound and may provide a platform for further in-depth studies of prodiginine biosynthesis.

## Introduction

Prodiginines are tripyrrolic red-colored secondary metabolites of microbial origin with highly valuable bioactivities, such as antibacterial, antitumor, immunosuppressive, and antimalarial activity (Han et al., 1998; Lapenda et al., 2014; Hassankhani et al., 2015). Within the prodiginine family, prodigiosin is one of the most prominent members (Williamson et al., 2006). Efficient production of prodigiosin is the prerequisite for further research on its biological effects and potential application of the compound.

The microbial biosynthesis offers a promising alternative to a laborious multi-step total chemical synthesis (Stankovic et al., 2014; Nisha et al., 2015). Prodigiosin is naturally synthesized from amino acid and acetate building blocks (Williamson et al., 2006) by different bacterial strains including species of *Serratia* (Thomson et al., 2000), *Hahella* (Jeong et al., 2005), and *Vibrio* (Allen et al., 2000), while *Streptomyces* sp. synthesize a mixture of other prodiginines (Williamson et al., 2006). Efforts aiming at the microbial prodigiosin production have so far primarily focused on the opportunistic human pathogen *Serratia marcescens* (Mahlen, 2011; Su et al., 2011; Chen et al., 2013; Stankovic et al., 2014). Besides safety reasons, heterologous production is highly attractive, as the use of well-established and genetically accessible expression hosts enables synthetic biology approaches to design novel biosynthetic pathways and optimize production levels. However, heterologous production of prodigiosin is demanding for several reasons. First, the prodigiosin pathway is in *S. marcescens* genetically encoded by 14 *pig* genes located in a 21 kb gene cluster (Harris et al., 2004). The corresponding biosynthesis is realized in a complex bifurcated pathway, producing precursors 2-methyl-3-amyl-pyrrole (MAP) and 4-methoxy-2,2′-bipyrrole-5-carbaldehyde (MBC) which are finally condensed to prodigiosin, as excellently reviewed by Williamson et al. (2006). MBC biosynthesis involves enzymes belonging to the PKS (polyketide synthase) and NRPS (non-ribosomal peptide synthase) family (Garneau-Tsodikova et al., 2006) that require specific enzymatic activation. Therefore, the large size of the gene cluster, the complexity of the biosynthesis pathway and not to neglect the antimicrobial activity of the final product, render heterologous prodigiosin production challenging.

So far, heterologous prodigiosin production at mg-scale could only be established in *Escherichia coli* by expressing the biosynthetic genes from *Hahella chejuensis* (Kwon et al., 2010). In addition, we have recently identified the GRAS (generally recognized as safe) certified strain *Pseudomonas putida* KT2440 as a promising prodigiosin producer (Loeschcke et al., 2013) in the context of validating a newly developed system for the transfer and expression of clustered genes (TREX). The prodigiosin biosynthesis encoding *pig* genes from *S. marcescens* were transferred to *P. putida* and integrated as TREX-*pig* transposon into the host chromosome. Subsequent T7 RNA polymerase-dependent, bidirectional expression of the *pig* genes resulted in prodigiosin biosynthesis. However, yields of these initial experiments were rather low with <1 mg/gDCW (g dry cell weight). Based on these findings, we aimed in this study at straightforward and enhanced *P. putida*-based prodigiosin production, employing unidirectional constitutive *pig* gene expression from a strong native host promoter.

## Results

### Construction of *P. putida* Prodigiosin Production Strains

In one of our previous studies, we could show that prodigiosin biosynthesis can in principle be implemented in *P. putida* strains by T7 RNA polymerase-dependent bidirectional transcription of *pig* genes (Loeschcke et al., 2013). Since product yields were comparatively low in these initial experiments, we employed here a new strategy, aiming at constitutive *pig* gene expression from a strong native *P. putida* promoter. We again applied random chromosomal integration of the unidirectionally oriented *pig* genes from *S. marcescens* into the *P. putida* chromosome. In contrast to our former experiments, however, insertion into highly transcribed genomic loci would install prodigiosin biosynthesis without the aid of T7 RNA polymerase which can be screened for by the corresponding red pigmentation phenotype.

**FIGURE 1:**
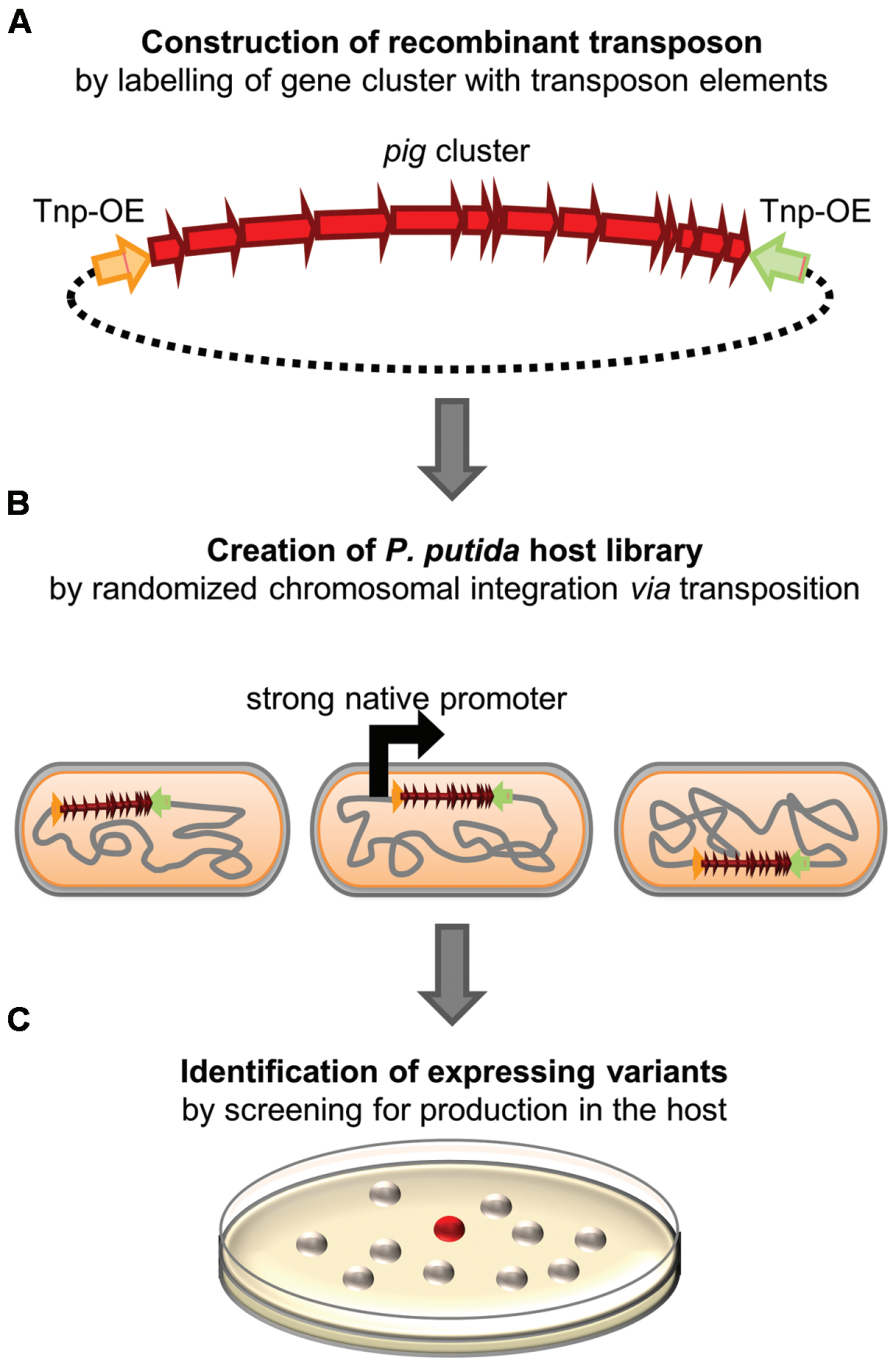
**Strategy for the construction of *Pseudomonas putida* prodigiosin production strains. (A)** The prodigiosin biosynthesis gene cluster from *Serratia marcescens* is flanked by Tn5 transposon elements, namely a transposase gene as well as transposon outside ends (Tnp-OE), thereby reconstituting a recombinant transposon. **(B)** The construct is used to create a library of *P. putida* clones carrying the *pig* gene cluster at different chromosomal loci. Thus, strongly transcribed chromosomal regions are statistically hit by transposition. **(C)** *P. putida* variants, in which strong *pig* gene transcription results in prodigiosin accumulation, are identified by red color.

**FIGURE 2:**
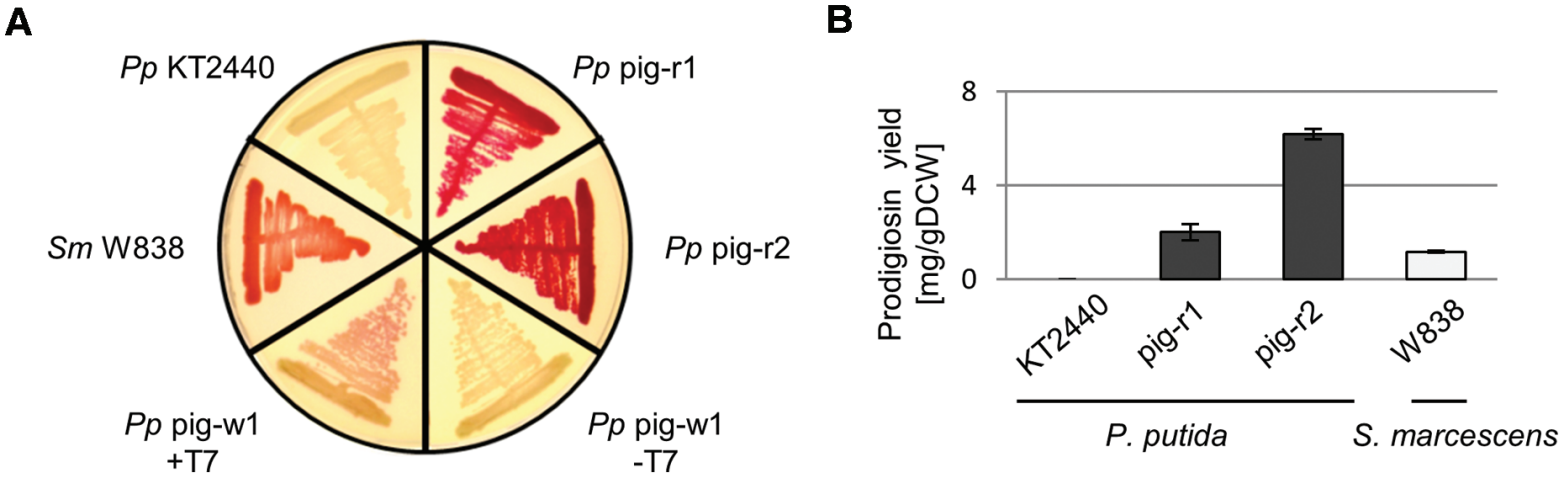
**Constitutive heterologous prodigiosin biosynthesis in *P. putida* pig-r1 and pig-r2. (A)** Color phenotypes of agar plate-grown *S. marcescens* (*Sm* W838) and *P. putida* (*Pp*) strains expressing *pig* genes. KT2440, wild-type; pig-r1 and pig-r2, constitutive prodigiosin producers; pig-w1, T7 RNA polymerase-dependent producer strain, in the absence (-T7) and presence (+T7) of T7 RNA polymerase. **(B)** Prodigiosin accumulation in bacterial cells grown under standard conditions in liquid medium (*P. putida*) or on agar plates (*S. marcescens*) for 24 h at 30°C. Extracts from cells were assayed photometrically for pigment content at 535 nm. Values represent means from three independent measurements. Error bars indicate the respective standard deviation.

To this end, we used the plasmid pTREX-pig which carries the complete prodigiosin gene cluster flanked by the DNA cassettes of the TREX system which include a gentamycin resistance gene as well as elements of transposon Tn5, enabling random chromosomal integration (Loeschcke et al., 2013). Since the ColE1 origin of this vector does not support vector replication in *P. putida*, cells carrying the TREX-*pig* transposon in their chromosome could be easily selected using gentamycin containing agar plates. A library of 1000 clones was screened after transposition of *pig* genes. By following the workflow depicted in **Figure 1**, we could readily identify two clones that showed constitutive, T7 RNA polymerase-independent prodigiosin production. Both clones, *P. putida* pig-r1 and *P. putida* pig-r2, exhibited an intense red color on agar plates, similar to that of the native producer *S. marcescens* (**Figure 2A**). The coloration of these strains was obviously more intense than in previously reported T7 RNA polymerase-dependent expression strains (*P. putida* pig-w1 + T7, **Figure 2A**), indicating a significantly higher prodigiosin production.

Red pigmentation of the two prodigiosin producing *P. putida* strains was observed both in early and late stages of colony growth on plates, indicating the *pig* gene expression being under control of strong and constitutively active promoters. Interestingly, plasmid rescue of parts of the TREX-*pig* transposon and determination of the insertion loci by sequencing (see Supplementary Figure S1A for details) revealed integration of the *pig* gene cluster into genes encoding ribosomal RNA for both *P. putida* strains (pig-r1: 23S RNA gene; pig-r2: 16S RNA gene). The *pig* genes were inserted in the same direction as the corresponding rRNA genes. Consequently, expression of the entire *pig* gene cluster is under control of the respective *P. putida* rRNA promoter (Supplementary Figure S1B, positions of *pig* genes relative to the promoter are marked). Like *E. coli, P. putida* harbors seven copies of rRNA operons with high sequence similarities of the genes and promoter regions. Here, the precise position of the *pig* gene cluster was not further determined. Notably, the mutations that were introduced by TREX-*pig* transposition into rRNA genes did not induce any apparent signs of cellular stress in the *P. putida* strains pig-r1 and pig-r2 (see below).

### Quantification of Prodigiosin Production in the Heterologous Host *P. putida*

In order to enable accurate analysis of prodigiosin in *P. putida*, we chemically synthesized the compound as a reference. Thus, a shortened synthesis route was developed based on published protocols (Trofimov et al., 1985; Hajipour et al., 1999; Dairi et al., 2006; Durchschein et al., 2010; Yu et al., 2012). The two biomimetic intermediates MAP and MBC were combined to prodigiosin in a final condensing reaction (see supplementary information 1 and Supplementary Figures S2–S4 for details on prodigiosin synthesis). However, overall yields were only moderate (12–13%), which underlines the need for an efficient biotechnological production process.

Pigments like prodigiosin allow for simple quantification in cell extracts *via* their spectral properties. We therefore determined the molar extinction coefficient to be ε_535_ [M^-1^cm^-1^] = 139,800 ± 5,100 in acidified ethanol (4% of 1 M HCl) using the chemically synthesized, pure prodigiosin. Purity was verified using quantitative ^1^H-NMR (qNMR; supplementary information 2). Prodigiosin production of *P. putida* strains in liquid medium could thus be assessed by preparing crude acidified ethanolic extracts from cells and subsequent determination of prodigiosin specific absorption. Under standard growth conditions in liquid medium, 2.0 ± 0.1 mg/gDCW and 6.2 ± 0.2 mg/gDCW were accumulated in *P. putida* strains pig-r1 and pig-r2, respectively (**Figure 2B**). Interestingly, in contrast to its intensely red phenotype on agar plates, *S. marcescens* strain W838 did not accumulate the pigment upon growth in liquid medium. Besides standard conditions, we tested different temperatures and media but did not observe prodigiosin production. In contrast, agar plate-grown cells of *S. marcescens* accumulated 1.2 ± 0.3 mg/gDCW.

### Influence of Temperature, Growth Medium, and Aeration on Prodigiosin Production

**FIGURE 3:**
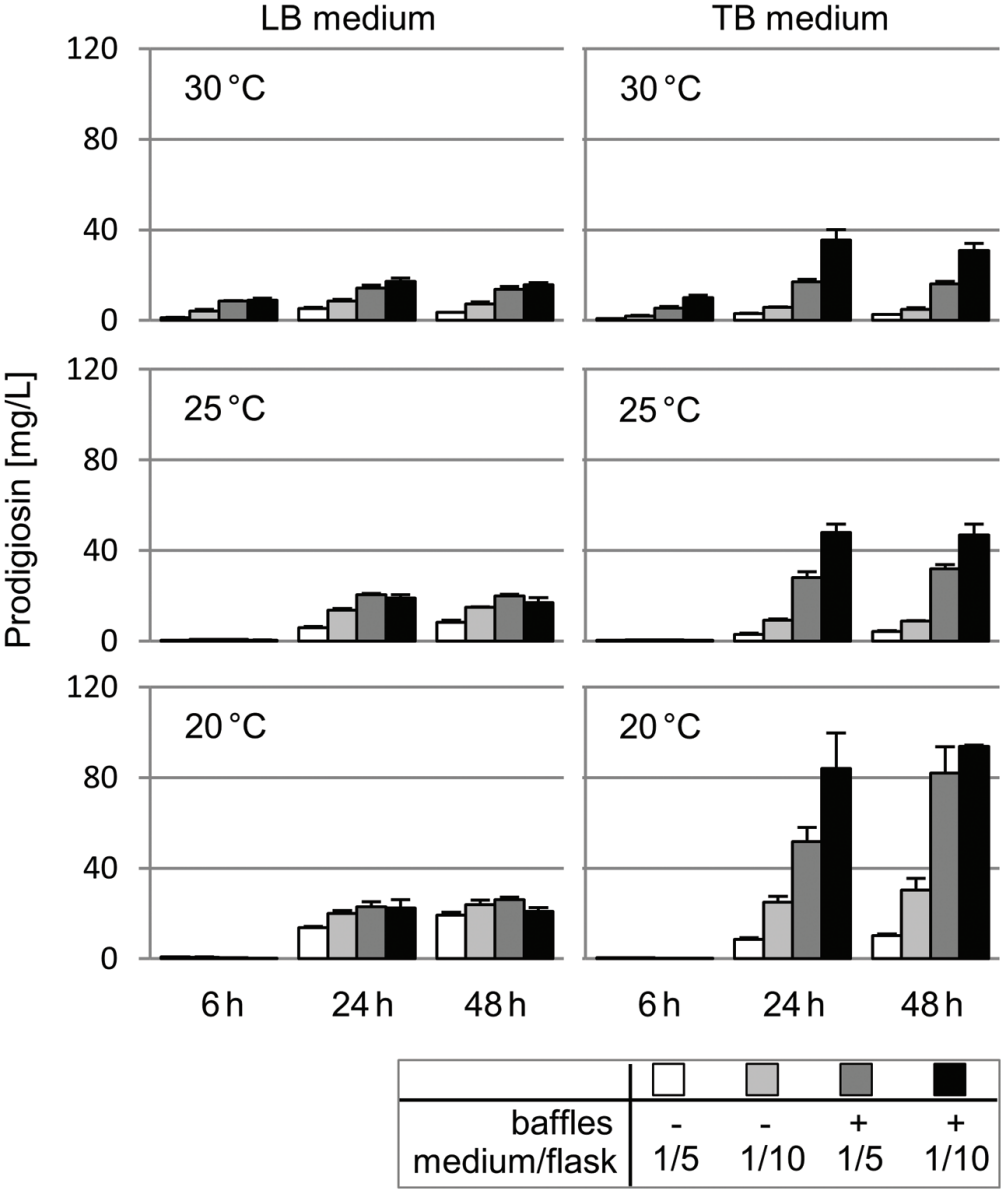
**Heterologous prodigiosin production in *P. putida* under different cultivation conditions.** Prodigiosin accumulation in *P. putida* pig-r2 when cultivated at different temperatures (30, 25, 20°C), in either LB or TB medium, in cultivation vessels filled with 1/5 or 1/10 of the flask capacity, in baﬄed and non-baﬄed flasks to implement different levels of aeration. Cultures were sampled after 6, 24 and 48 h to prepare cell extracts for photometric determination of pigment content at 535 nm. Values represent means from three independent measurements. Error bars indicate the respective standard deviation.

In order to estimate the potential of the producer strain *P. putida* pig-r2 for prodigiosin production, common cultivation parameters including temperature (30, 25, 20°C), medium composition (LB, TB medium) and aeration (1/5 or 1/10 filling volumes of the total flask capacity, in non-baﬄed and baﬄed flasks) were comparatively evaluated with respect to pigment accumulation. Cultures were sampled in the early (after 6 h) and late logarithmic growth phase (after 24 h) as well as in the stationary phase (after 48 h) to measure prodigiosin content in acidified ethanolic extracts. Results are presented as amount of prodigiosin [mg] in 1 L of bacterial culture broth (**Figure 3**).

Prodigiosin production at 30°C in LB medium varied significantly under different aeration conditions, established by use of lower filling levels and/or baﬄed flasks, reaching titers from 1.2 ± 0.1 to 17 ± 1 mg/L. Cultivation in rich TB medium further broadened the production range to 0.8 ± 0.1 to 36 ± 5 mg/L. In both, LB and TB, prodigiosin accumulation increased over the logarithmic growth phase (6–24 h), and remained unchanged or decreased slightly in the stationary growth phase. Remarkably, especially with TB-grown cells, improved aeration resulted in higher prodigiosin titers.

At 25°C, overall higher pigment levels than at 30°C were observed, with highest values of 48 ± 4 mg/L (for 1/10 TB medium in a baﬄed flask, at 24 h). Changes in media composition and increased aeration produced a similar profile as observed at 30°C. However, in contrast to growth at 30°C, no substantial prodigiosin production was observed at the early stage of cultivation (6 h). Cultivation at 20°C basically resulted in a reproduction of the profile observed at 25°C, but with peak prodigiosin titers again increased by factor 1.7–2.0. Maximal prodigiosin production of 84 ± 16 and 94 ± 1 mg/L was thus observed at 20°C, in 1/10 TB medium in a baﬄed flask, after 24 and 48 h, respectively. A maximal volumetric productivity of 3.5 ± 0.7 mg/L/h was obtained under the same growth conditions at 20°C, in 1/10 TB medium in a baﬄed flask, after 24 h (see Supplementary Table S1 for details on prodigiosin titers and volumetric productivity).

Since changes in cultivation conditions also affect cell growth, it is worth underlining that at 25 and 20°C, low prodigiosin levels per liter at 6 h are in fact a result of decreased prodigiosin accumulation per cell mass and not only due to lower cell densities than at 30°C. In contrast, higher prodigiosin titers in TB compared to LB directly reflect a significantly higher cell mass in the culture and not enhanced prodigiosin yields per cell mass. Highest specific prodigiosin production per cell mass were reached after 48 h at 20°C in 1/10 LB (13 ± 1 mg/gDCW) and TB medium (14 ± 1 mg/gDCW), in non-baﬄed and baﬄed flasks, respectively (see Supplementary Table S1 for details on specific product yields expressed in mg/gDCW).

The following experimental observations may be worth mentioning: (i) Prodigiosin was extracted from cell pellets harvested by centrifugation; however, the supernatant still remained slightly red-colored. This minor fraction which was left behind after centrifugation was not further considered in the assessment of improved production conditions. (ii) Notably, cell pellets were often observed to show light, only faint red color and appeared coated with a thick deeply red-colored layer. It may therefore be stated that prodigiosin apparently accumulated in structures with a sedimentation behavior slightly different to *P. putida* cells. (iii) In addition, prodigiosin production levels remained well-reproducible in *P. putida*, also after several subsequent cultivations of *P. putida* strains on agar plates.

### *P. putida* Tolerates Prodigiosin Production

**FIGURE 4:**
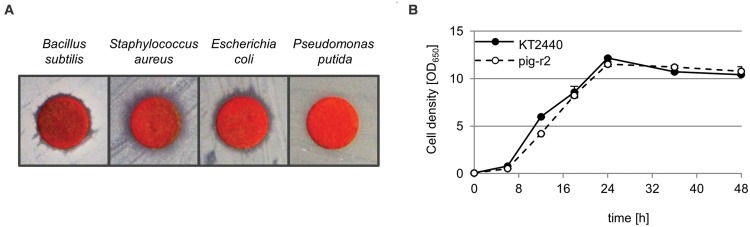
**Tolerance of *P. putida* toward the antibiotic prodigiosin. (A)** Disk diffusion assay with prodigiosin (50 μg) on *Bacillus subtilis, Staphylococcus aureus, Escherichia coli*, and *P. putida*. Clear zones in bacterial lawns around disks indicate prodigiosin-related growth inhibition. **(B)** Growth of prodigiosin producing *P. putida* pig-r2 and wild-type KT2440 under optimal production conditions as determined previously (at 20°C, in 1/10 TB medium in baﬄed flasks). Cell density was determined photometrically (OD_650_) in intervals over 48 h. Data points represent means from three independent measurements. Error bars indicate the respective standard deviation.

Prodigiosin is known to have antibiotic activity; however, its activity against *Pseudomonas* species is discussed controversially (Samrot et al., 2011; Gulani et al., 2012; Priya et al., 2013; Sumathi et al., 2014). Reasons may include different experimental set-ups and strain specific characteristics. We therefore analyzed susceptibility of the here used *P. putida* strain KT2440 in a simple disk diffusion assay in direct comparison to *Bacillus subtilis* and human pathogen *Staphylococcus aureus* that were previously reported as susceptible (Samrot et al., 2011; Gulani et al., 2012; Priya et al., 2013), and in comparison to *E. coli*, an alternative heterologous host for prodigiosin production. The zones of inhibition observed for *B. subtilis* and *S. aureus* confirmed previous reports (**Figure 4A**). Similar observations could be made for *E. coli*. In contrast, *P. putida* did not show growth inhibition when exposed to prodigiosin.

Remarkably, intracellular prodigiosin production in *P. putida* strains pig-r1 and pig-r2 did not lead to any apparent signs of stress like heterogeneity in clone size or color, as can typically be observed in the context of the production of toxic compounds (Beuttler et al., 2011). Instead, clones showed a stable uniform phenotype. The tolerance against endogenously produced prodigiosin is further clearly demonstrated by comparing cell growth of *P. putida* wild-type and prodigiosin production strain pig-r2 under maximal production conditions (**Figure 4B**). During the logarithmic growth phase, we detected a slightly decelerated growth of the producer in comparison to the wild-type. However, cells reached exactly the same cell density in the stationary growth phase (after 24 h). These findings corroborate that *P. putida* provides a highly robust platform organism for heterologous prodigiosin production.

### Efficient Extraction and Purification of Prodigiosin from *P. putida*

**FIGURE 5:**
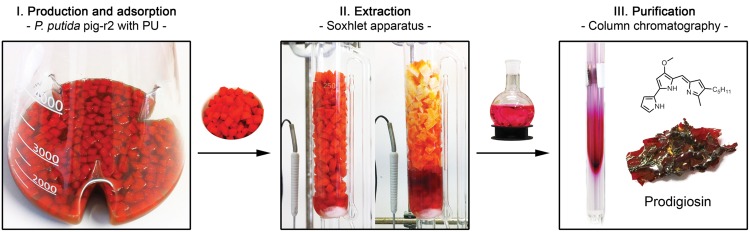
**Extraction and purification of prodigiosin from *P. putida*.** *P. putida* pig-r2 was cultivated together with polyurethane (PU) foam cubes in the culture broth that function as adsorbent for hydrophobic prodigiosin. Foam cubes were recovered by sieving and continuously extracted applying a Soxhlet apparatus. The prodigiosin-containing extract was further purified *via* column chromatography to yield prodigiosin as a solid.

To establish a novel easy-to-perform protocol for isolation of prodigiosin based on the presented robust *P. putida* production platform, *P. putida* pig-r2 was cultivated at larger scale in 500 mL growth medium at the above described optimal prodigiosin production conditions. Based on the fact that prodigiosin is a hydrophobic molecule and can thus be adsorbed to hydrophobic surfaces when in an aqueous environment (Song et al., 2006; Juang and Yeh, 2014), a straightforward strategy for *in situ* product recovery from *P. putida* cells and culture supernatant was developed. It was evaluated whether hydrophobic polyurethane (PU) added as foam cubes to the cultivation vessel could serve as adsorbents for prodigiosin (**Figure 5**, step I). Remarkably, the foam cubes did not only adsorb the small fraction of prodigiosin accumulating in the supernatant, but seemed to facilitate prodigiosin secretion by continuously binding of the extracellular pigment, resulting in almost uncolored cells and culture medium (visualized in Supplementary Figure S5). These observations were corroborated by spectrophotometric quantification of the remaining cell-bound prodigiosin which revealed titers below 1 mg/L.

The foam cubes could be easily separated from the culture broth by sieving and were subjected to a continuous extraction with acidified ethanol using a Soxhlet apparatus (**Figure 5**, step II). This extraction method enables effective extraction of prodigiosin from PU and can be followed visually as fully extracted PU is left uncolored. After removal of the solvent, prodigiosin was further purified applying two-phase extraction with water and dichloromethane. The organic layer containing the product was dried yielding 126 ± 18 mg of crude prodigiosin extract as a red solid from 1 L culture.

Crude extracts were analyzed in comparison to chemically synthesized prodigiosin by UV-VIS spectroscopy, HPLC and HRMS (Supplementary Figures S6–S8), verifying the correct properties and structure of the extracted compound. Quantification *via* the extinction coefficient revealed the portion of prodigiosin in crude extracts to be only ca. 35% of crude extract total mass which was additionally verified by qNMR. Assuming full recovery of prodigiosin, cultures with PU foam cubes thus accumulated about 47 mg/L prodigiosin. Surprisingly, in the same up-scaled cultivation set-up, prodigiosin titers without PU were only 24 ± 8 mg/L. PU-based extraction therefore resulted in increased prodigiosin titers and at the same time enabled more effective and easy isolation of prodigiosin from the cultivation broth (see Supplementary Table S2 for a direct comparison). Prodigiosin could be further purified from crude extracts *via* column chromatography using dichlormethane as eluent (**Figure 5**, step III). The hereby yielded 56 ± 7 mg solid material (obtained from 1 L culture) contained 84% prodigiosin, as analyzed *via* qNMR.

## Discussion

*Pseudomonas putida* KT2440 was chosen as prodigiosin production host for several reasons. Ideal prerequisites include that it is a certified GRAS strain, it is fully sequenced, and its genetic manipulation is well-established. The bacterium has thus been employed as a heterologous host for diverse complex natural product biosyntheses (Loeschcke and Thies, 2015), most prominently in the context of myxobacterial compounds (Wenzel et al., 2005; Li et al., 2010; Chai et al., 2012). The strain offers specific advantages, rendering challenging biosyntheses of natural products like prodigiosin possible: (i) It harbors a phosphopantetheinyl transferase (PPTase) with broad substrate specificity (Gross et al., 2005). Enzymes of this family are required for functional PKS/NRPS expression, because they activate peptidyl carrier protein (PCP) as well as acyl carrier protein (ACP) domains, which also occur in enzymes PigG and PigH of the prodigiosin pathway, respectively (Garneau-Tsodikova et al., 2006), by attaching the phosphopantetheinyl moiety to them. It was previously described that the *pig* gene cluster most probably contains its own PPTase encoding gene (*pigL*) activating the ACP in PigH, but the PCP of PigG is activated by another PPTase in the native producer *S. marcescens* (Sunaga et al., 2004). In the heterologous host *P. putida*, the activation of this domain is therefore accomplished by an intrinsic PPTase. (ii) *P. putida* DNA has a relatively high GC content (61.5%), which exactly matches that of the *pig* genes from *S. marcescens* and hence provides an ideal background for correct protein translation. (iii) *P. putida* exhibits tolerance toward various antibiotic substances. In the presented work, we could show that *P. putida* KT2440 tolerates prodigiosin concentrations from intrinsic production of up to 94 mg/L and can sustain production stably over many generations without apparent costs on cellular vitality. Potentially, efficient eﬄux systems which are typically activated by the presence of xenobiotics in *P. putida* (Fernández et al., 2009; Simon et al., 2014) prevent the intracellular accumulation of prodigiosin. Furthermore, it has been described that several *P. putida* strains including KT2440 produce outer membrane vesicles in response to stress including the presence of xenobiotics (Kobayashi et al., 2000; Baumgarten et al., 2012a,b; Choi et al., 2014). These vesicles increase the cell surface hydrophobicity and thus enhance biofilm formation (Baumgarten et al., 2012a). The hydrophobic cell surface and vesicle structures could potentially provide an extracellular storage room for hydrophobic compounds such as prodigiosin. This might explain our observation of only slightly red-colored cell pellets coated with a deep red layer and why prodigiosin could be captured by adsorption to PU foam cubes. However, further experiments are needed to confirm these hypotheses.

For high-level prodigiosin production in *P. putida*, we found high aeration, rich medium and low temperature to be favorable for product accumulation in shake flask cultivation. As prodigiosin titers (mg/L) correlated with cell densities, the positive effect of high aeration is largely due to improved energy balance and thus growth of the aerobic bacterium under these conditions (Duetz et al., 2000). It might be further speculated that aeration has a direct influence on prodigiosin biosynthesis. This may be deduced from a tendency to higher specific prodigiosin yields per cell mass (mg/gDCW) at high-aeration conditions. The positive effect of rich medium on prodigiosin production per liter is a direct result of increased cell-density but might additionally be due to enhanced precursor supply including, e.g., amino acids proline and serine. The ideal temperature for a heterologous production process is usually unpredictable and must be determined experimentally. We found low temperature to enhance product titers significantly. Similar tendencies were observed before in the context of myxochromide S production using *P. putida* (Wenzel et al., 2005). However, this can certainly not be generalized since there are also studies reporting the opposite (Chai et al., 2012).

Previously, different studies have reported on prodigiosin production using the pathogenic natural producer *S. marcescens* with yields at gram-scale, e.g., ca. 39 g/L (Giri et al., 2004), ca. 2.4 g/L (Su et al., 2011), and 15.6 g/L (Chen et al., 2013). Successful heterologous prodigiosin production has so far been shown in *Erwinia carotovora* (Thomson et al., 2000) and *E. coli* (Kwon et al., 2010). Although absolute yields were not in the focus of these studies prodigiosin production in *E. coli* can be estimated by the given data (A_534_/OD_600_) to be in the range of 10 mg/gDCW. Using *P. putida* KT2440, we show here with 94 mg/L the highest level of heterologous prodigiosin production reported so far.

For the *in situ* recovery of prodigiosin from *P. putida* culture broth, we could demonstrate the usefulness of PU foam as an adsorbent which binds the product without the need to break the producing cells or extract the entire supernatant, similar to previously described applications of PU in the context of other metabolites (Heyes et al., 2003). This low-tech and low-cost approach may pose an interesting alternative to commonly applied resins for *in situ* product recovery such as HP-20 or X-5 (Kim et al., 1999; Bae et al., 2001; Wang et al., 2004; Song et al., 2006). Our effective and easy two-step purification protocol initially yielded a crude extract with up to 35% of product content which could be fully recovered in a second purification step yielding 56 mg of 84% pure compound from 1 L bacterial culture. Notably, in contrast to HPLC and MS data, which are commonly used in literature for the indication of purity and quantification of prodigiosin in extracts, only qNMR or the use of a qNMR-based extinction coefficient can give correct quantitative information. This is highlighted in this study by the fact that HPLC measurements at one wavelength revealed only traces of impurities (<3%), whereas qNMR-based analysis determined about 65% of impurities in the prodigiosin crude extract (Supplementary Figure S7).

Previously, we have demonstrated that the TREX expression system is a useful tool for establishing biosynthesis of secondary metabolites in heterologous hosts based on T7 RNA polymerase-dependent gene expression (Loeschcke et al., 2013). In that context, applying TREX enabled the identification of *P. putida* KT2440 as promising heterologous producer of prodigiosin. In the present study, we show another useful application, relying on native host promoters which were targeted by chance as transposon Tn5 inserts the gene cluster at random positions in the bacterial chromosome. We thereby identified promoters of ribosomal RNA genes to be particularly suitable for the expression of the 21 kb *pig* gene cluster in *P. putida* and thus might prove useful for heterologous expression of other target genes in the bacterium as well.

The strategy of random chromosomal integration of a biosynthetic gene cluster may be applicable in versatile contexts of screening and production purposes in a broad range of bacterial hosts. However, the following limitations should be considered: (i) The approach presented here is only applicable for unidirectional gene clusters like the *pig* cluster whose expression can be realized by the activity of a single promoter. (ii) Transcriptional terminators existing in a gene cluster could hamper expression by the host RNA polymerase. If these aspects pose limitations for complete transcription, implementation of convergent expression from two promoters flanking the gene cluster and use of T7 RNA polymerase which appears to ignore bacterial termination sites (Studier, 1972; Studier and Moffatt, 1986; Widenhorn et al., 1988; Arvani et al., 2012), as applied in the TREX system, may offer an alternative solution. (iii) Success in finding variants that exhibit strong expression of pathway genes by a native host promoter fully depends on an exercisable screening method, which was here the simple visual detection of color formation. In the case of other metabolites, parallelizable sophisticated analytical methods will be required for the identification of producers in transposon libraries.

In summary, we report here effective biosynthetic prodigiosin production by expression of the *S. marcescens* pathway genes in the heterologous host *P. putida* as well as straightforward product recovery. The proposed strategy may further be applicable for the production and isolation of other compounds with biomedical relevance. Moreover, prodigiosin biosynthesis now being available in the well-established host renders it accessible to genetic manipulation and further in-depth studies.

## Materials and Methods

### Bacterial Strains and Culture Conditions

*Escherichia coli* strains DH5*α* (Hanahan, 1983), S17-1 (Simon et al., 1983), and BL21 (Studier and Moffatt, 1986; applied for cloning, conjugation, and assessment of antibacterial activity of prodigiosin, respectively) as well as *S. aureus* (strain collection of the Institute of Molecular Enzyme Technology) were cultivated on LB-agar plates or under constant shaking (120 rpm) at 37°C in LB (lysogeny broth) liquid medium (LB medium (Luria/Miller), Carl Roth^®^, Karlsruhe, Germany: 10 g/L trypton, 5 g/L yeast extract, 10 g/L sodium chloride. Standard conditions for *P. putida* wild type KT2440 (Nelson et al., 2002) and prodigiosin production strains (*P. putida* pig-r1 and *P. putida* pig-r2) were cultivation on LB-agar plates or in liquid LB medium under constant shaking (120 rpm) at 30°C. *S. marcescens* W838 (DSM No. 12487) and *B. subtilis* 168 (Burkholder and Giles, 1947) were cultivated on LB-agar plates or in liquid LB medium under constant shaking (120 rpm) at 30°C. Antibiotics were added to the culture medium to the following final concentrations [μg/mL]: *E. coli*: 100 (ampicillin), 10 (tetracycline); *P. putida*: 25 (gentamicin).

### Construction of *P. putida* Prodigiosin Production Strains

The *pig* gene cluster from *S. marcescens* W838 was integrated into the chromosome of *P. putida* KT2440 *via* transposition. The plasmid pTREX-pig (Loeschcke et al., 2013), carrying the *pig* gene cluster and transposon Tn5 elements, was transformed into *E. coli* S17-1 and further transferred to *P. putida* by conjugation as previously described (Loeschcke et al., 2013). In *P. putida*, pTREX-pig is a suicide vector. Positive selection for transposon mutants was conducted by using LB medium supplemented with gentamicin. In addition to the selection antibiotic, 25 μg/mL irgasan were added to prevent *E. coli* growth. Prodigiosin producing strains were identified on agar plates by their red color.

### Prodigiosin Production in *P. putida*

Standard conditions for prodigiosin production were defined as application of 100 mL non-baﬄed flasks with 1/10 (filling volume/flask capacity) LB medium, incubated at 30°C with constant shaking (120 rpm). Precultures (grown under standard conditions) were used to inoculate production cultures starting with a cell density of OD_650_ = 0.05.

Different cultivation parameters were evaluated applying baﬄed or non-baﬄed 500 mL flasks, LB or TB medium (Terrific-Broth, modified, Carl Roth^®^, Karlsruhe, Germany: 12 g/L Casein, enzymatically digested, 24 g/L yeast extract, 9.4 g/L dipotassium phosphate, 2.2 g/L monopotassium phosphate, 4 mL/L glycerol), 1/5 or 1/10 filling/flask ratio and incubation at 30, 25, or 20°C cultivation temperature.

Production of prodigiosin for extraction and purification was conducted using 5 L baﬄed flasks filled with 500 mL TB medium and a surface covering layer (5 g) of PU foam cubes (Bornewasser, Göllheim, Germany: softpur, 25 kg m^-3^ density, 4 kPa compression hardness), each approximately 1 cm^3^. Cultures were incubated with shaking at 20°C for 48 h until harvesting of foam cubes.

### Quantification of Prodigiosin Production in *P. putida*

Cell material of *S. marcescens* W838, *P. putida* pig-r1 or *P. putida* pig-r2 corresponding to an OD_650_ = 1 was harvested by centrifugation and extracted with 1 mL acidified ethanol (4% v/v of 1 M HCl). The extracts were cleared by centrifugation. Prodigiosin was quantified spectrophotometrically based on a molar extinction coefficient determined as ε_535_ = 139,800 ± 5,100 *M*^-1^cm^-1^ in acidified ethanol. Production was determined as titer by calculating prodigiosin mass per liter culture (mg/L), as volumetric productivity taking production time into account (mg/L/h) and as product accumulation per cell mass (mg/gDCW). In order to determine the latter using relative prodigiosin quantities and cell densities, a calibration curve was used to define the correlation of OD_650_ = 1 to 0.717 mgDCW.

### Determination of Chromosomal Integration Loci of *pig* Genes *via* Plasmid Rescue

As described above, the generated prodigiosin production strains carry the *pig* genes as a Tn5-based transposon from pTREX-pig in their chromosome. The recombinant transposon comprises the *pig* genes, elements of the TREX cassettes and vector elements from pUC19, including an ampicillin resistance gene and the *E. coli* ColE1 origin of replication, enabling a plasmid rescue strategy: genomic DNA of *P. putida* pig-r1 and *P. putida* pig-r2 was isolated using DNeasy Blood & Tissue Kit (Qiagen, Hilden, Germany) and hydrolyzed with *Mlu*I (*P. putida* pig-r1) or *Nco*I (*P. putida* pig-r2). Hydrolyzed DNA was ligated with T4 DNA ligase and afterward used for transformation of *E. coli* DH5*α*. Selection with ampicillin enabled fishing and amplification of plasmid molecules containing parts of the transposon and parts of *P. putida* genomic DNA. Plasmid DNA was isolated and sequenced using oligonucleotide Seq-out-OER (5′-ACGGGAAAGGTTCCGTTCAGG-3′) as primer. Chromosomal integration loci of the *pig* cluster were identified employing *Pseudomonas* genome database (http://www.pseudomonas.com/; Winsor et al., 2011).

### Assessment of *P. putida*’s Tolerance toward Prodigiosin

Antibiotic activity of externally added prodigiosin was monitored using a modified disk diffusion assay (Bauer et al., 1966). Cells from an overnight culture were harvested and dissolved in sterile saline (0.9% w/v NaCl) to an OD_580_ = 0.15. This cell suspension was used to inoculate Mueller-Hinton-agar plates with sterile cotton buds. 5 μl of chemically synthesized prodigiosin, dissolved in ethanol (10 mg/mL), were applied to sterile disks of Whatman-Paper (5 mm in diameter), the disks were dried on air and applied to the inoculated Mueller-Hinton agar plates. The antibiotic effect of prodigiosin was estimated after overnight incubation of plates at the optimal growth temperature of each strain (*B. subtilis, S. aureus, E. coli*, and *P. putida*) according to Leibniz Institute DSMZ (German Collection of Microorganisms and Cell Cultures). Equally treated disks with ethanol were used as negative control.

Cell growth of the prodigiosin production strain *P. putida* pig-r2 was compared to the wild-type KT2440 under optimal production conditions (500 mL baﬄed shake flasks, filled with 1/10 TB medium, incubated at 20°C; ca. 90 mg/L prodigiosin production) by measuring cell density (OD_650_) over 48 h.

### Extraction and Purification of Prodigiosin

The extraction of prodigiosin from the PU foam cubes of 500 mL cultures was performed with a Soxhlet extractor. The red-colored foam cubes were recovered from the cell culture by sieving, wrung out and charged into a 250 mL Soxhlet apparatus fitted with a reflux condenser and a 500 mL round bottom flask filled with 300 mL of acidified ethanol (4% v/v, 1 M HCl) or diethyl ether. The sample was continuously extracted by heating with the chosen solvent. For the extraction of prodigiosin from the cells of 500 mL cultures without PU, 50 mL portions of broth were centrifuged (15′, 4°C, 16,000 × *g*) to pellet the cells. Collected pellets (from 100 mL broth) were then extracted twice with 10 mL acidified ethanol. Cell debris was removed by centrifugation and extracts were combined. In both procedures (with or without PU), the solvent of extracts was removed under reduced pressure by rotatory evaporation and the residual material was extracted with water and dichloromethane (3 mL × 30 mL) to remove water soluble impurities. The combined organic layers were washed with brine (2 mL × 20 mL) and dried over MgSO_4_. The solvent was removed under reduced pressure yielding the prodigiosin extract as a red solid. The crude extracts were analyzed by UV-VIS spectroscopy, HPLC, HRMS, and compared to chemically synthesized prodigiosin (Supplementary Figures S6–S8). The quantity of prodigiosin was verified by quantitative ^1^H-NMR with 4-methoxyphenol as internal standard (Supplementary information 2).

Preparative flash column chromatography for further purification of the crude extract was performed using silica gel 60 (particle size 0.040-0.063 mm, 230-240 mesh) and dichloromethane providing prodigiosin as a deep red solid. Dichloromethane was distilled prior to use. Purification was monitored by thin layer chromatography (TLC) on pre-coated plastic sheets (Polygram^®^ SIL G/UV254, Macherey–Nagel) with detection by ultraviolet irradiation at 254 nm and treatment with an acidic solution of *p*-anisaldehyde followed by brief heating with a heat gun.

## Author Contributions

AL, TD, K-EJ, TC, and JP designed the research experiments; AD, AK, JH-H, ST, and VS conducted the experiments; AL, AK, AD, and TD wrote the manuscript.

## Conflict of Interest Statement

The authors declare that the research was conducted in the absence of any commercial or financial relationships that could be construed as a potential conflict of interest.
