# Supplementary material for: Efficient recombinant production of prodigiosin in Pseudomonas putida
Source: Front Microbiol. 2015 Sep 15;6:972. doi: 10.3389/fmicb.2015.00972 (PMC4569968; doi:10.3389/fmicb.2015.00972)
Supplement: Supplementary file 1 [file Data_Sheet_1.PDF]

Supplementary Material

Efficient recombinant production of prodigiosin in *Pseudomonas putida*

Andreas Domröse[1,†], Andreas S. Klein[2,†], Jennifer Hage-Hülsmann[1], Stephan Thies[1], Vera Svensson[1], Thomas Classen[2], Jörg Pietruszka[2,3], Karl-Erich Jaeger[1,3] , Thomas Drepper[1]*, Anita Loeschcke[1,*]

†equally contributed, *corresponding authors

Affiliations:

1 Institute of Molecular Enzyme Technology, University Düsseldorf, Research Center Jülich, Germany
2 Institute of Bioorganic Chemistry, University Düsseldorf, Research Center Jülich, Germany
3 Institute of Bio- and Geosciences, Research Center Jülich, Germany

Correspondence:
Thomas Drepper, Anita Loeschcke
t.drepper@fz-juelich.de
a.loeschcke@fz-juelich.de

Institute of Molecular Enzyme Technology
Research Center Jülich,
Stetternicher Forst
D-52426 Jülich, Germany

Content

I Generation of *P. putida* prodigiosin production strains

Supplementary Figure S1	Determination of *pig* gene insertion locus in *P. putida* pig-r1 and pig-r2	S–2

II Chemical synthesis of prodigiosin as a reference

Supplementary information 1	Chemical synthesis of prodigiosin	S–4
Supplementary Figure S2	Scheme for chemical synthesis of prodigiosin	S–8
Supplementary Figure S3	^{1}H-NMR analysis of chemically synthesized prodigiosin	S–9
Supplementary Figure S4	^{13}C-NMR analysis of chemically synthesized prodigiosin	S–10
Supplementary information 2	Determination of extinction coefficient of prodigiosin in acidified ethanol	S–11

III Prodigiosin production in *P. putida*

Supplementary Table S1	Prodigiosin production in *P. putida* (mg/L), (mg/L/h), (mg/gDCW)	S–12
Supplementary Figure S5	Adsorption of prodigiosin by polyurethane foam in culture broth	S–13
Supplementary Table S2	Comparison of extraction procedures with and without polyurethane foam	S–14

IV Analytical comparison of biotechnologically produced prodigiosin to the chemical reference

Supplementary Figure S6	UV/VIS spectra	S–15
Supplementary Figure S7	HPLC chromatograms	S–15
Supplementary Figure S8	HRMS spectra	S–16

Supplementary references S–17

I Generation of *P. putida* prodigiosin production strains

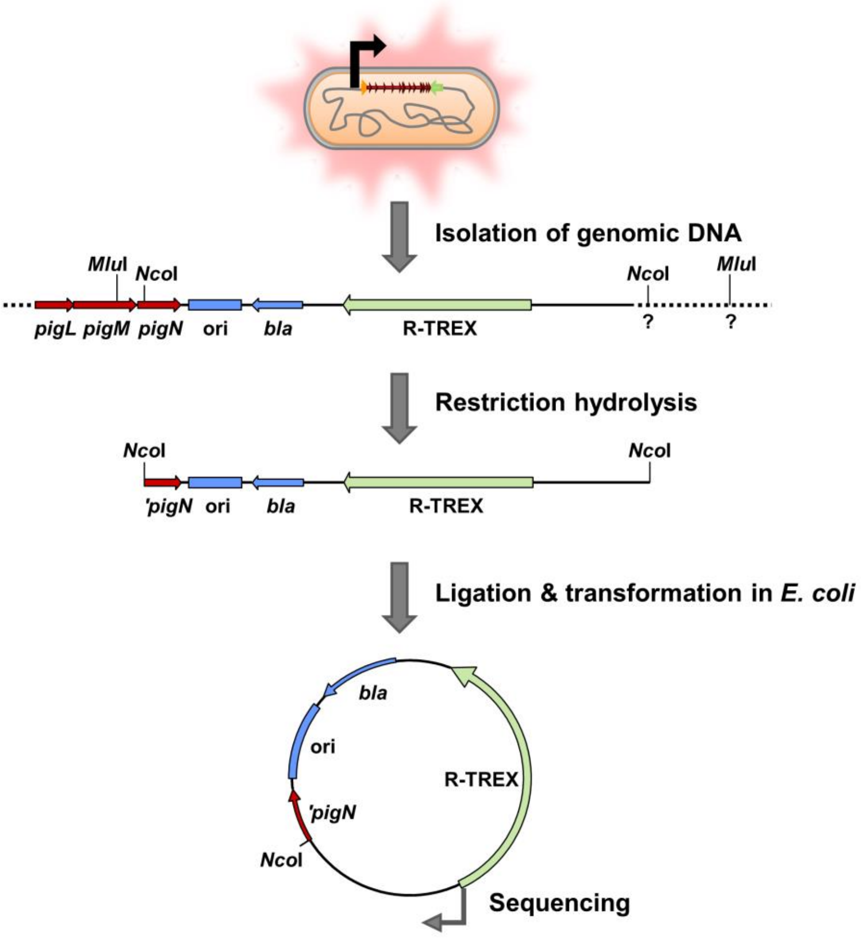

Supplementary Figure S1A | Determination of the insertion locus of *pig* genes in the chromosome of *P. putida* pig-r1 and pig-r2.
Plasmid rescue: As a first step, genomic DNA was isolated from prodigiosin producing *P. putida* strains. The chromosome harbors the recombinant transposon encompassing the *pig* genes together with elements (ori, *bla*) from vector pTREXpig and TREX cassettes. Enzymatic restriction hydrolysis with endonucleases recognizing sites occurring within the genes *pigM* or *pigN* was applied to yield fragments that comprise the vector elements and a portion of genomic DNA. Ligation produced circular replicative plasmids that can be transformed into *E. coli* ('plasmid rescue'). The plasmid DNA was used for determination of the insertion locus by sequencing.

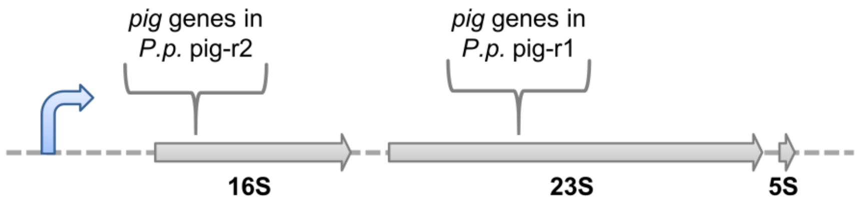

Supplementary Figure S1B | Determination of the insertion locus of *pig* genes in the chromosome of *P. putida* pig-r1 and pig-r2.

Chromosomal insertion loci: *P. putida* KT2440 harbors seven highly similar copies of ribosomal RNA operons, each spanning ca. 6 kb. They are all arranged in the sequence 16S > 23S > 5S rRNA (schematically depicted: promotor as blue arrow, rRNA genes in grey), some additionally containing small tRNA genes (not indicated). Chromosomal insertion loci of *pig* genes in strains *P. putida* pig-r1 and pig-r2 are schematically marked: In *P. putida* pig-r1, the TREX-*pig* transposon was inserted into a 23S rRNA gene, while in strain pig-r2, *pig* genes were inserted into a 16S rRNA gene. The exact copy of rRNA operon was not determined in either case.

II Chemical synthesis of prodigiosin as a reference

Supplementary information 1: Chemical synthesis of prodigiosin

General methods forchemical synthesis procedures

All chemical reagents were purchased from *Sigma-Aldrich, Steinheim,* Germany, *Alfa Aesar, Karlsruhe, Germany* or *TCI-Europe, Zwijndrecht, Belgium*. Preparative column chromatography was performed using silica gel 60 (particle size 0.040-0.063 mm, 230-240 mesh). Petroleum ether (PE), ethyl acetate (EtOAc), diethyl ether and dichloromethane were distilled prior to use. All other chemicals and solvents were used as purchased without further purification.

Reactions were monitored by thin layer chromatography (TLC) on pre-coated plastic sheets (Polygram® SIL G/UV254, Macherey–Nagel) with detection by ultraviolet irradiation at 245 nm or treatment with a basic solution of $KMnO_4$, an acidic solution of *p*-anisaldehyde or ceric ammonium molybdate followed by brief heating with a heat gun.

Microwave syntheses were performed using a CEM Discover® reactor.

GC-MS analysis was performed on a HP 6890 series gas chromatograph (Hewlett Packard) equipped with a HP 6890 series injector and a split injection system, fitted with a HP-5 ms column (30 m x 0.25 mm, 0.25 µm, Agilent Technologies) and coupled with a mass selective detector 5973 mass spectrometer. The temperatures of the injector and the detector were fixed at 250 °C and 230 °C, respectively. Helium was used as the carrier gas at 0.57 bar. Mass spectra were collected in the electron impact mode at 70 eV. The column temperature was initially 60 °C for 1 min, then raised to 185 °C at a rate of 15 °C min^{-1}, subsequently raised to 280 °C at a rate of 120 °C min^{-1} and maintained at that temperature for 5 min.

^{1}H- and ^{13}C-NMR spectra were recorded on an Advance/DRX 600 nuclear magnetic resonance spectrometer (Bruker) at ambient temperature in $CDCl_3$ at 600 and 151 MHz, respectively. The chemical shifts are given in ppm relative to tetramethylsilane [^{1}H: $\delta(SiMe_4)$ = 0.00 ppm] as an internal standard or relative to the solvent [^{13}C: $\delta(CDCl_3)$ = 77.16 ppm].

HPLC separations were performed on a Dionex system equipped with a pump with a gradient mixer and devolatilizer included a WPS-3000TSL autosampler and a DAD-3000 UV-detector using a Chiralpak® IA column (250 x 4.6 mm, 5 µm, Chiral Technologies). For all measurements the column temperature was 25 °C, the flow rate was 0.5 mL min^{-1} and 465 nm was used as the detection wavelength. A mixture of heptane and 2-propanol (80:20) was used as solvent.

HRMS (ESI-FTMS) measurements were performed by the Zentralinstitut für Engineering, Elektronik und Analytik of Forschungszentrum Jülich.

Names are in accordance with the IUPAC nomenclature, the atom numbering of the molecules is only used for NMR assignment.

Experimental procedures for the preparation of compounds 1-9 (Supplementary Figure S2)

(*E*/*Z*)-Octan-2-oneoxime (**1**) was synthesized according to the published methods of Durchschein et al. (2010) and Hajipour et al. (1999).

Method 1: In a sealed microwave vessel 2-octanone (**2**) (157 µL, 1 mmol), grounded hydroxylamine hydrochloride (140 mg, 2 mmol) and silica gel 60 (particle size 0.040–0.063 mm, 230–240 mesh) were mixed. The reaction was carried out at 90 °C, 150 W within 2 min in a CEM microwave. After cooling to room temperature 5 mL of 1 N HCl was added and the mixture was filtered through a pad of Celite and extracted with EtOAc (3 x 5 mL). The light brown extract was

dried over $MgSO_4$ and the solvent was evaporated providing compound **1** (0.9 mmol, 91%). The oxime was used without further purification in the following experiment.

Method 2: A mixture of 2-octanone (**2**) (3.70 mL, 23.4 mmol), grounded hydroxylamine hydrochloride (2.44 g, 35.1 mmol) and pyridine (1.5 mL, 18.6 mmol) in ethanol (20 mL) was refluxed for 2 h. The completion of the reaction was monitored by TLC using a $KMnO_4$ solution for staining. The reaction was extracted with EtOAc (3 x 25 mL) and the organic phase was washed several times with 1 N HCl and water. The light brown organic phase was dried over $MgSO_4$ and the solvent was evaporated providing compound **1** (3.28 g, 22.9 mmol, 98%). The oxime was used without further purification in the following experiment.

The NMR, IR and MS data are in accordance to literature (Beauchemin et al., 2008; Durchschein et al., 2010).

R_f = 0.55 & 0.72 (PE:EtOAc = 80:20 + TEA (1% v/v))
MS (EI, 70 eV): m/z = 143 [$(M)^+$], 129, 114, 101, 86, 73
E/*Z* ratio determined by NMR: 3:1
Major:
^{1}H-NMR (600 MHz, $CDCl_3$): δ [ppm] = 0.84-0.91 (m, 3H, 8-H), 1.22-1.37 (m, 6H, 5, 6, 7-H), 1.45-1.55 (m, 2H, 4-H), 1.91 (s, 3H, 1-H), 2.21 (t, $^3J_{3,4}$ = 7.8 Hz, 2H, 3-H), 9.84 (brs, 1H, 10-OH)
Minor:
^{1}H-NMR (600 MHz, $CDCl_3$): δ [ppm] = 0.84-0.91 (m, 3H, 8-H), 1.22-1.37 (m, 6H, 5, 6, 7-H), 1.45-1.55 (m, 2H, 4-H), 1.92 (s, 3H, 1-H), 2.40 (t, $^3J_{3,4}$ = 7.8 Hz, 2H, 3-H), 9.84 (brs, 1H, 10-OH)
^{13}C-NMR (151 MHz, CDCl3): δ [ppm] = 14.05 (8-C-8), 19.67 (C-1), 22.53 (C-7), 26.22 (C-6), 28.84 (C-5), 31.55 (C-4), 35.65 (C-3), 159.31 (C-2)
IR (atr-film): $\tilde{\upsilon}$ [cm^{-1}] = 3237, 3217, 3121, 2955, 2926, 2859, 1665, 1585, 1522, 1456, 1368, 1335, 1314, 1294, 1260, 1246, 1221, 1206, 1177, 1111, 1074, 1026, 939, 889, 862, 827, 754, 725, 650, 611

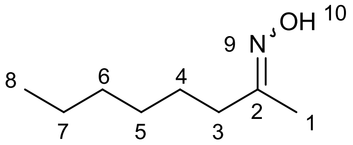

2-Methyl-3-amyl-pyrrole (MAP) (**3**) was synthesized according to a published procedure of Trofimov et al. (1985). A mixture of (*E*/*Z*)-octan-2-oneoxime (**1**) (2.18 g, 15.2 mmol), potassium hydroxide (4.27 g, 76 mmol), DMSO (20 mL) and water (200 µL) was heated for 30 min at 125 °C in a two-neck round-bottom flask fitted with a reflux condenser under nitrogen atmosphere. A solution of 1,2-dichloroethane (2.10 mL, 26.6 mmol) in DMSO (5 mL) was added dropwise over a period of 1 h. An additional amount of potassium hydroxide (4.266 g, 76 mmol) was added carefully within 15 min and thereafter a second portion of 1,2-dichloroethane (2.10 mL, 26.6 mmol) in DMSO (5 mL) was added dropwise within the next 1 h. After stirring for an additional 1 h the reaction was poured into ice water and extracted with diethyl ether (3 x 15 mL). The combined organic layers were dried over $MgSO_4$ and the solvent was removed under reduced pressure. Chromatography on silica gel with PE:EtOAc (99:1) + TEA (1% v/v) providing compound **3** (1.13 g, 7.5 mmol, 49%) as a light orange oil. The reaction was monitored by TLC using a $KMnO_4$ solution for staining.

The NMR, IR and MS data are in accordance to literature (Williamson et al., 2005).

R_f = 0.76 (PE:EtOAc = 80:20 + TEA (1% v/v))
^{1}H-NMR (600 MHz, $CDCl_3$): δ [ppm] = 0.89 (t, $^3J_{10,9}$ = 6.9 Hz, 2H, 10-H), 1.28-1.37 (m, 4H, 8, 9-H), 1.53 (m, 2H, 7-H), 2.18 (s, 3H, 11-H), 2.37 (t, $^3J_{6,7}$ = 7.7 Hz, 2H, 6-H), 6.01 (dd,

$^3J_{4,5}$ = 2.7 Hz, $^4J_{4,1}$ = 2.6 Hz, 1H, 4-H), 6.58 (dd, $^3J_{5,4}$ = 2.7 Hz, $^4J_{5,1}$ = 2.6 Hz, 1H, 5-H), 7.70 (brs, 1H, 1-NH)
^{13}C-NMR (151 MHz, $CDCl_3$): δ [ppm] = 11.03 (C-11), 14.13 (C-10), 22.64 (C-9), 25.88 (C-6), 31.03 (C-7), 31.80 (C-8), 108.86 (C-4), 114.80 (C-5), 119.77 (C-3), 123.16 (C-2)
MS (EI, 70 eV): m/z = 151 [(M)$^+$], 94, 80, 67
IR (atr-film): $\tilde{\upsilon}$ [cm^{-1}] = 3381, 2957, 2856, 1464, 1378, 1108, 901, 832, 711, 667

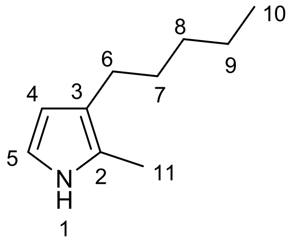

(5-Bromo-3-methoxy-pyrrol-2-ylidenemethyl)-diethylamine (**4**) was synthesized according to the published methods of Dairi et al. (2006) and Haynes et al. (2008). The reaction was performed under an inert atmosphere of dry nitrogen in a Schlenk flask fitted with a reflux condenser. To a solution of phosphorus oxybromide (12.67 g, 44.2 mmol) in dichloromethane (60 mL) at 0 °C was added dropwise a solution of diethylformamide (**5**) (5.91 mL, 53.0 mmol) in dichloromethane (20 mL). The mixture was stirred for 30 min at 0 °C. The solution turns bright yellow and a white solid is precipitating. A solution of 4-methoxy-3-pyrrolin-2-one (**6**) (2.0 g, 17.7 mmol) in dichloromethane (15 mL) was added dropwise and the reaction was slowly warmed to room temperature and heated at 40 °C for 2 h, 1 h at 50 °C (reflux) and thereafter cooled to room temperature and stirred for 16 h. The dark brown reaction mixture was poured into 200 mL ice water and the aqueous solution was neutralized with 1 N NaOH. EtOAc (80 mL) was added and the precipitated phosphorus salt was filtered off. The reaction was extracted with EtOAc (3 x 80 mL); the combined organic layers were washed with brine (2 x 50 mL) and water and dried over $MgSO_4$. The solvent was removed under reduced pressure and the crude product was purified by flash chromatography on silica gel with PE:EtOAc (85:15) + TEA (1% v/v) providing compound **4** (3.31 g, 12.8 mmol, 72%) as a light brown oil, which solidified on standing. The reaction was monitored by TLC using an acidic solution of *p*-anisaldehyde for staining.
The NMR, IR and MS data are in accordance to literature (Dairi et al., 2006; Haynes et al., 2008).

R_f = 0.35 (PE:EtOAc = 80:20 + TEA (1% v/v))
^{1}H-NMR (600 MHz, $CDCl_3$): δ [ppm] = 1.27 (t, 3H, $^3J_{12,11}$ = 7.1 Hz, 12-H), 1.30 (t, $^3J_{12,11}$ = 7.1 Hz, 3H, 12-H), 3.39 (q, $^3J_{11,12}$ = 7.1 Hz, 2H, 11-H), 3.76 (s, 3H, 8-H), 4.12 (q, $^3J_{11,12}$ = 7.1 Hz, 2H, 11-H), 5.59 (s, 1H, 5-H), 6.99 (s, 1H, 9-H)
^{13}C-NMR (151 MHz, $CDCl_3$): δ [ppm] = 12.50 (12-C), 14.61 (12-C), 44.54 (11-C), 51.12 (11-C), 57.99 (8-C), 96.47 (4-C), 120.81 (2-C), 133.72 (5-C), 138.59 (9-C), 165.32 (3-C)
MS (EI, 70 eV): m/z = 258 [(M)$^+$], 243, 229, 215, 201, 185, 179, 163, 149, 135, 123, 108, 92, 78, 56
IR (atr-film): $\tilde{\upsilon}$ [cm^{-1}] = 2976, 2936, 1626, 1528, 1447, 1408, 1381, 1351, 1291, 1263, 1195, 1140, 1115, 1072, 995, 906, 856, 818, 737, 681, 668
Mp: 44-45 °C

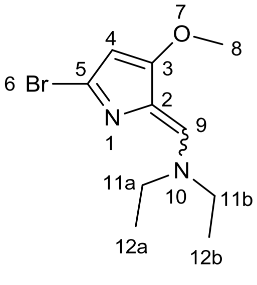

***tert*-Butyl 5′-formyl-4′-methoxy-1*H*,1′*H*-2,2′-bipyrrole-1-carboxylate (Boc-MBC)** (**7**) was synthesized under an inert atmosphere of dry nitrogen in a Schlenk flask fitted with a reflux condenser. Dioxane and water were previously degassed by bubbling dry nitrogen. To a solution of tetrakis(triphenylphosphine)palladium(0) (11 mg, 5 mol%) in toluene (40 µL) at room temperature was added dropwise a solution of *N*-Boc-pyrrole-2-boronic acid MIDA ester (**8**) (75 mg, 0.23 mmol) and compound **4** (50 mg, 0.19 mmol) in water/dioxane (10:90) (730 µL) followed by the addition of sodium carbonate (61 mg, 0.58 mmol). The mixture was heated at 95 °C (reflux) and became bright yellow. After 1 h a solution of potassium carbonate (70 µL, 6 M) was added and heating was continued for 2 h. Thereafter the reaction mixture was stirred at 60 °C for 16 h. The dark orange reaction mixture was poured into 8 mL ice water and the solution was neutralized with 2 N HCl. The precipitated salt was filtered off and washed with small amounts of water, acetone, dichloromethane and diethyl ether. The reaction was extracted with diethyl ether (3 x 10 mL); the combined organic layers were dried over $MgSO_4$ and the solvent was removed under reduced pressure. The crude product was purified by flash chromatography on silica gel with PE:EtOAc (80:20) + TEA (1% v/v) providing compound **7** (42 mg, 0.15 mmol, 77%) as a light orange solid. The reaction was monitored by TLC using an acidic solution of *p*-anisaldehyde for staining.
The NMR, IR and MS data are in accordance to literature (Aldrich et al., 2010).

R_f = 0.28 (PE:EtOAc = 80:20 + TEA (1% v/v))
^{1}H-NMR (600 MHz, $CDCl_3$): δ [ppm] = 1.62 (s, 9H, 9, 10, 11-H), 3.89 (s, 3H, 7′-H), 6.08 (d, $^4J_{4',1'}$ = 2.9 Hz, 1H, 4′-H), 6.25 (dd, $^3J_{4,3}$ = 3.5 Hz, $^3J_{4,5}$ = 3.5 Hz, 1H, 4-H), 6.67 (dd, $^3J_{3,4}$ = 3.6 Hz, $^4J_{3,5}$ = 1.8 Hz, 1H, 3-H), 7.33 (dd, $^3J_{5,4}$ = 3.4 Hz, $^4J_{5,3}$ = 1.8 Hz, 1H, 5-H), 9.54 (s, 1H, 8′-H), 10.70 (s, 1H, 1′-H)
^{13}C-NMR (151 MHz, $CDCl_3$): δ [ppm] = 27.93 (C-9, 10, 11), 57.91 (C-7′), 85.80 (C-8), 94.78 (C-4′), 111.49 (C-4), 116.87 (C-3), 118.28 (C-2′), 124.53 (C-5), 126.00 (C-2), 130.20 (C-5′), 149.70 (C-3′), 157.60 (C-6), 174.40 (C-8′)
MS (EI, 70 eV): m/z = 190 [$(MBC)^+$], 175, 146, 131, 116, 63
IR (atr-film): $\tilde{\upsilon}$ [cm^{-1}] = 3206, 3156, 3013, 2978, 1744, 1597, 1545, 1504, 1477, 1460, 1445, 1429, 1389, 1366, 1304, 1285, 1254, 1231, 1190, 1171, 1159, 1140, 1082, 1059, 1015, 918, 878, 8429, 820, 768, 733, 593, 685, 640
Mp: 146-148 °C

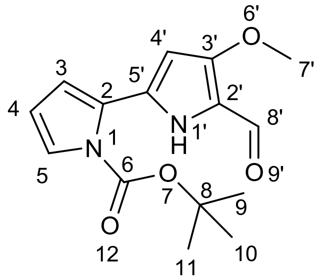

4-Methoxy-5-((5-methyl-4-pentyl-2*H*-pyrrol-2-ylidene)methyl)-1*H*,1′*H*-2,2′-bipyrrole (prodigiosin) (**9**) was synthesized according to a published method of Yu et al. (2012) under an inert atmosphere of dry nitrogen in a Schlenk flask. To a solution of compound **7** (150 mg, 0.52 mmol) and compound **3** (94 mg, 0.62 mmol) in dry dichloromethane (20 mL) was added dropwise phosphorus oxychloride (482 µL, 5.2 mmol) at 0 °C. The deep red reaction mixture was stirred for 2 h at room temperature. Thereafter the reaction was quenched by adding TEA (725 µL) and extracted with water and dichloromethane (3 x 50 mL). The combined organic layers were washed with brine (2 x 20 mL) and dried over $MgSO_4$. The solvent was removed under reduced pressure and the crude product was purified by flash chromatography on silica gel

with dichloromethane providing compound **9** (87 mg, 0.27 mmol, 52%) as a deep red solid. The reaction was monitored by TLC using an acidic solution of *p*-anisaldehyde for staining.

R_f = 0.30 (dichloromethane)
^{1}H-NMR (600 MHz, $CDCl_3$): δ [ppm] = 0.90 (t, $^3J_{10'',9''}$ = 6.9 Hz, 3H, 10″-H), 1.22-1.39 (m, 4H, 8″, 9″-H), 1.54 (tt, $^3J_{7'',6''}$ = 7.5 Hz, $^3J_{7'',8''}$ = 7.5 Hz, 2H, 7″-H), 2.39 (t, $^3J_{6'',7''}$ = 7.6 Hz, 2H, 6″-H), 2.54 (s, 3H, 11″-H), 3.99 (s, 3H, 7′-H), 6.07 (d, $^4J_{4'',1''}$ = 1.7 Hz, 1H, 4′-H), 6.32-6.36 (m, 1H, 4-H), 6.65-6.69 (m, 1H, 4″-H), 6.91 (ddd, $^3J_{3,4}$ = 4.0 Hz, $^4J_{3,5}$ = 2.4 Hz, $^5J_{3,1}$ = 1.2 Hz, 1H, 3-H), 6.94 (s, 1H, 8′-H), 7.20-7.24 (m, 1H, 5-H), 12.55 (s, 1H, 1-H), 12.64-12.77 (m, 2H, 1′, 1″-H) (Supplementary Figure S3)
^{13}C-NMR (151 MHz, $CDCl_3$): δ [ppm] = 12.43 (C-11″), 14.04 (C-10″), 22.49 (C-9″), 25.31 (C-6″), 29.79 (C-7″), 31.42 (C-8″), 58.71 (C-7′), 92.82 (C-4′), 111.71 (C-4), 115.97 (C-8′), 117.02 (C-3), 120.68 (C-2′), 122.23 (C-2), 125.12 (C-5″), 126.88 (C-5), 128.36 (C-4″), 128.45 (C-3″), 146.90 (C-2″), 147.68 (C-5′), 165.74 (C-3′)
(Supplementary Figure S4)
HRMS (ESI-FTMS, positiv Ion): calculated for $C_{20}H_{26}N_3O$ $(M + H)^+$ = 324.4408; found 324.20696 ± 0.23181
IR (atr-film): $\tilde{\upsilon}$ [cm^{-1}] = 3150, 3102, 3071, 2955, 2922, 2855, 1628, 1605, 1578, 1545, 1508, 1449, 1412, 1387, 1356, 1339, 1329, 1261, 1252, 1200, 1138, 1082, 1067, 1043, 1026, 997, 989, 959, 891, 835, 808, 785, 777, 745, 737, 718, 698, 648, 623
Mp: 144-146 °C
ε_{535} [M^{-1}cm^{-1}] = 139,800 ± 5,100

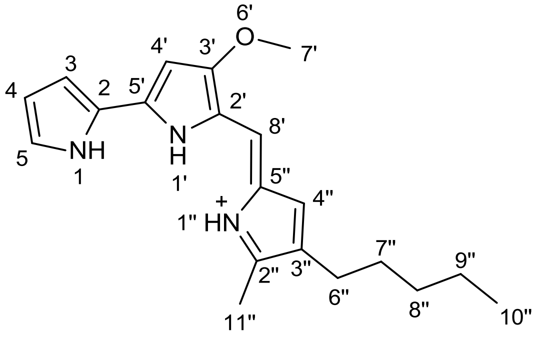

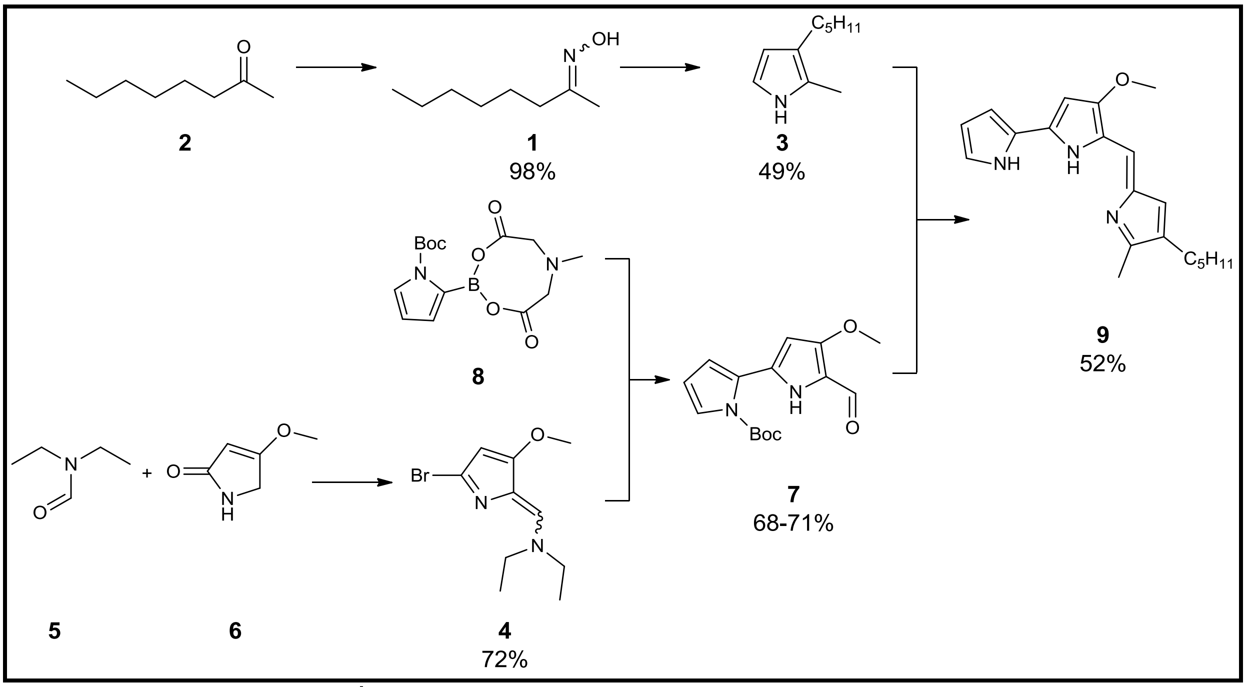

Supplementary Figure S2 | Chemical synthesis of prodigiosin (9).

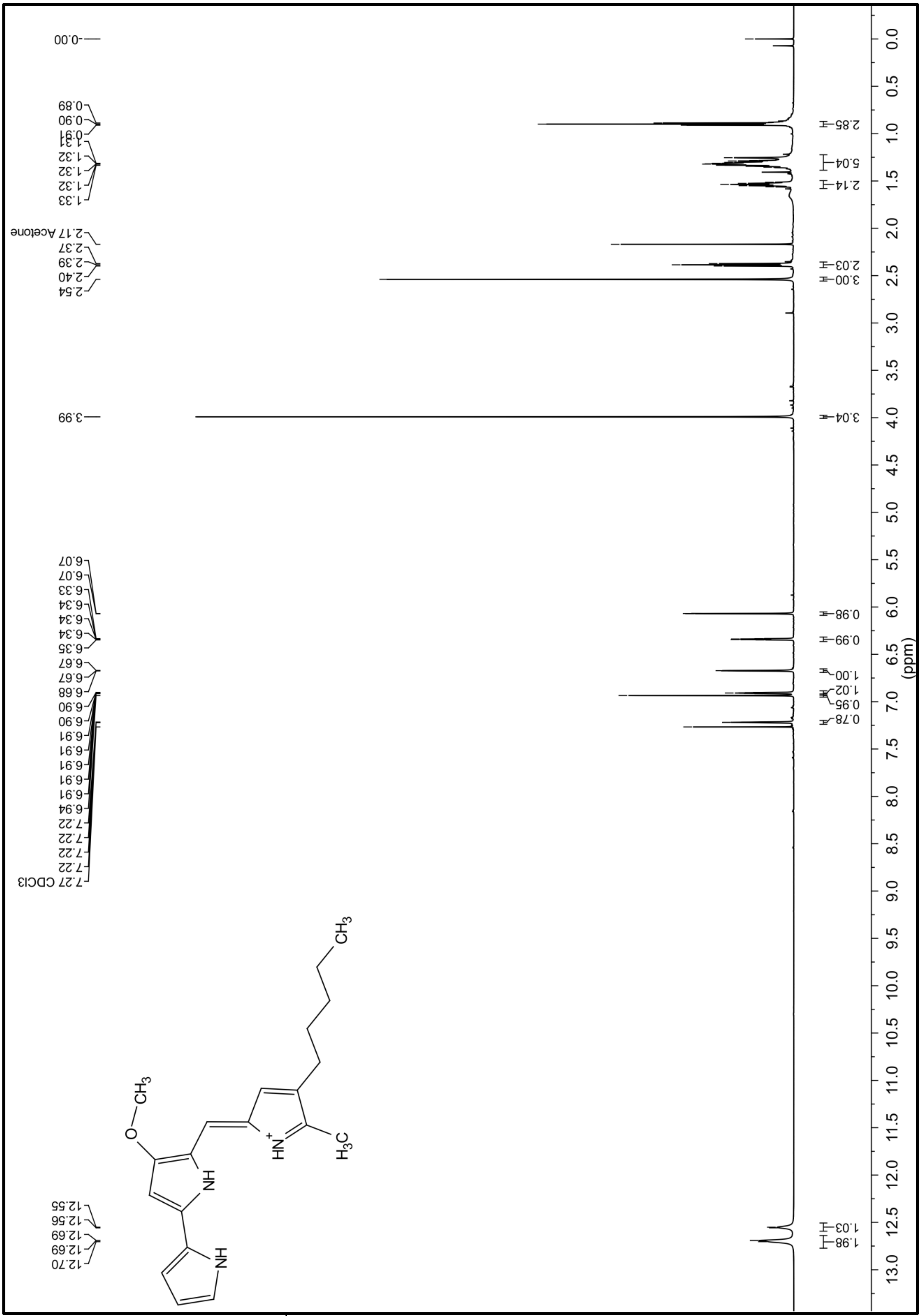

Supplementary Figure S3 | ^{1}H-NMR of prodigiosin (9) in $CDCl_3$.

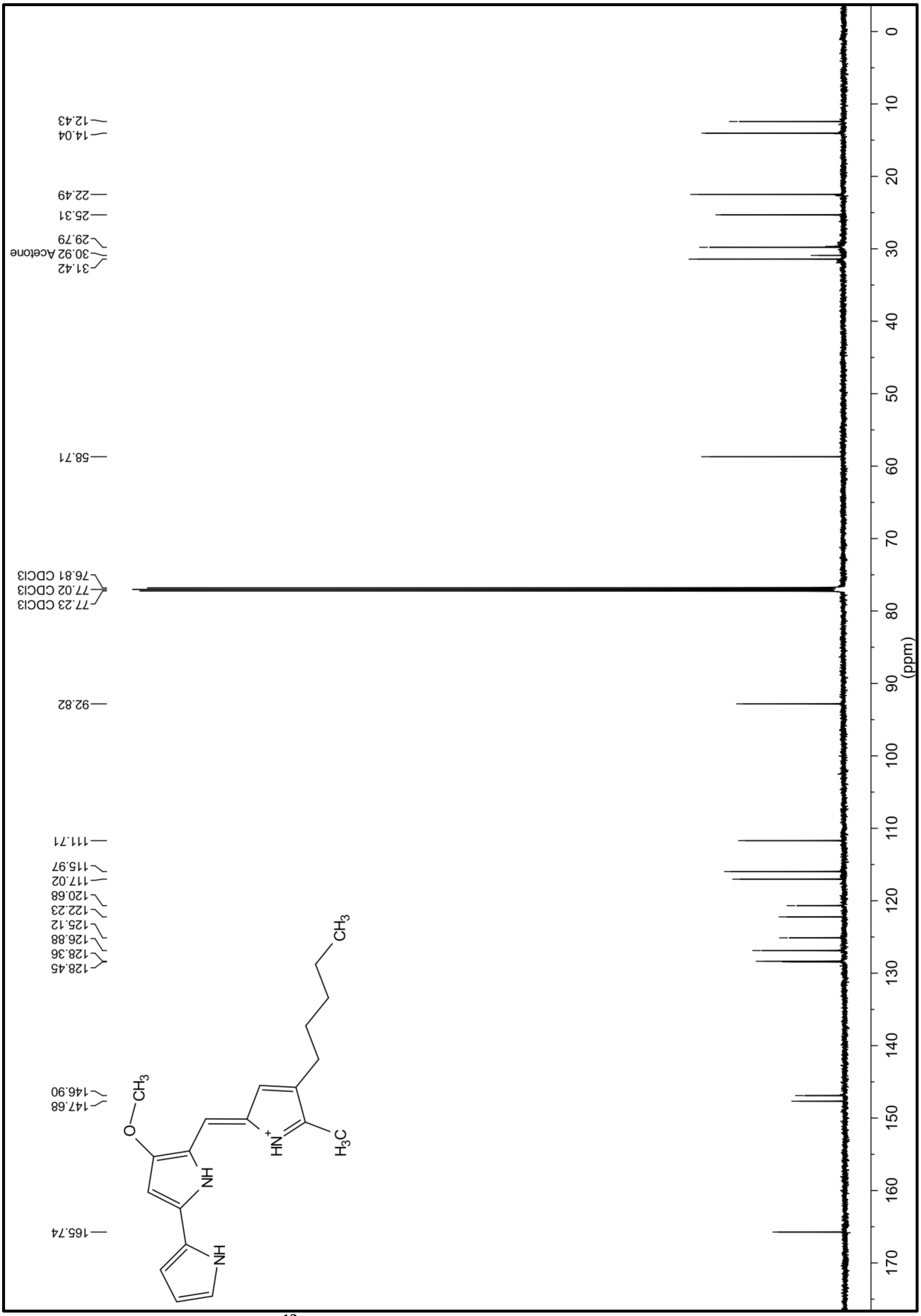

Supplementary Figure S4 | ^{13}C-NMR of prodigiosin (9) in $CDCl_3$.

Supplementary information 2: Determination of the molar extinction coefficient of prodigiosin in acidified ethanol

The molar extinction coefficient of prodigiosin was determined from the slope by plotting the absorption of the pigment versus the concentration at the absorption maximum (535 nm) in acidified ethanol (4% v/v of 1 N HCl). The purity of the pigment was verified by quantitative ^{1}H-NMR with 4-methoxyphenol as internal standard using following equation:

$$\boldsymbol{m_{Prod} = m_{Std} \times \frac{N_{Std}}{N_{Prod}} \times \frac{M_{Prod}}{M_{Std}} \times \frac{I_{Prod}}{I_{Std}}}$$

Prod = product; Std = internal standard; m = weight; N = number of absorbing protons; M = molecular weight; I = signal area

The signal areas were determined by the integrals of the methoxy groups of 4-methoxyphenol (3.76 ppm) and prodigiosin (3.99 ppm).

Supplementary Table S1 | Prodigiosin production in *P. putida*, expressed as prodigiosin titer (mg/L), volumetric productivity (mg/L/h) and specific product yield per cell mass (mg/gDCW).
Bacteria were cultivated at different temperatures (30, 25, 20 °C), in either LB or TB medium, in cultivation vessels filled with 1/5 or 1/10 of the flask capacity, in non-baffled and baffled flasks (indicated with - and +) to implement different levels of aeration. Cultures were sampled after 6, 24 and 48 h to prepare cell extracts for photometric determination of pigment content at 535 nm. Values represent means from three independent measurements (mv) with standard deviations (sd). A gradient color code indicates high values in red in order to visualize trend patterns.

mg/L		LB												TB											
		6 h				24 h				48 h				6 h				24 h				48 h			
		1/5 -	1/10 -	1/5 +	1/10 +	1/5 -	1/10 -	1/5 +	1/10 +	1/5 -	1/10 -	1/5 +	1/10 +	1/5 -	1/10 -	1/5 +	1/10 +	1/5 -	1/10 -	1/5 +	1/10 +	1/5 -	1/10 -	1/5 +	1/10 +
30 °C	mv	1.21	4.19	8.58	9.01	5.26	8.53	14.25	17.25	3.52	7.24	13.68	15.81	0.76	1.92	5.38	10.12	3.07	5.79	17.02	35.48	2.61	4.83	16.07	30.92
	sd	0.09	0.68	0.24	0.78	0.50	0.84	1.29	1.39	0.13	0.90	1.30	0.91	0.11	0.30	0.85	0.97	0.12	0.23	1.04	4.54	0.07	0.74	1.24	3.14
25 °C	mv	0.36	0.76	0.73	0.42	5.97	13.58	20.52	18.94	8.36	14.99	19.90	16.91	0.38	0.46	0.46	0.30	2.97	9.14	28.05	48.02	4.17	8.85	31.93	46.87
	sd	0.02	0.02	0.01	0.03	0.41	0.83	0.51	1.48	0.81	0.09	0.79	2.27	0.01	0.02	0.04	0.02	0.51	0.70	2.62	3.70	0.40	0.15	1.77	4.76
20 °C	mv	0.73	0.70	0.37	0.28	13.75	20.03	22.97	22.41	19.21	23.91	26.03	20.87	0.39	0.35	0.13	0.10	8.62	24.97	51.66	83.94	10.23	30.29	82.01	93.74
	sd	0.14	0.15	0.01	0.04	0.51	1.19	2.22	3.62	1.35	1.94	1.12	1.77	0.04	0.03	0.03	0.02	0.69	2.49	6.36	15.78	0.64	5.20	11.57	0.55

mg/L/h		LB												TB											
		6 h				24 h				48 h				6 h				24 h				48 h			
		1/5 -	1/10 -	1/5 +	1/10 +	1/5 -	1/10 -	1/5 +	1/10 +	1/5 -	1/10 -	1/5 +	1/10 +	1/5 -	1/10 -	1/5 +	1/10 +	1/5 -	1/10 -	1/5 +	1/10 +	1/5 -	1/10 -	1/5 +	1/10 +
30 °C	mv	0.20	0.70	1.43	1.50	0.22	0.36	0.59	0.72	0.07	0.15	0.28	0.33	0.13	0.32	0.90	1.69	0.13	0.24	1.48	0.71	0.05	0.10	0.33	0.64
	sd	0.02	0.11	0.04	0.13	0.02	0.04	0.05	0.06	0.00	0.02	0.03	0.02	0.02	0.05	0.14	0.16	0.01	0.01	0.19	0.04	0.00	0.02	0.03	0.07
25 °C	mv	0.06	0.13	0.12	0.07	0.25	0.57	0.86	0.79	0.17	0.31	0.41	0.35	0.06	0.08	0.08	0.05	0.12	0.38	1.17	2.00	0.09	0.18	0.67	0.98
	sd	0.00	0.00	0.00	0.01	0.02	0.03	0.02	0.06	0.02	0.00	0.02	0.05	0.00	0.00	0.01	0.00	0.02	0.03	0.11	0.15	0.01	0.00	0.04	0.10
20 °C	mv	0.12	0.12	0.06	0.05	0.57	0.83	0.96	0.93	0.40	0.50	0.54	0.43	0.07	0.06	0.02	0.02	0.36	1.04	2.15	3.50	0.21	0.63	1.71	1.95
	sd	0.02	0.03	0.00	0.01	0.02	0.05	0.09	0.15	0.03	0.04	0.02	0.04	0.01	0.01	0.01	0.00	0.03	0.10	0.27	0.66	0.01	0.11	0.24	0.01

mg/gDCW		LB												TB											
		6 h				24 h				48 h				6 h				24 h				48 h			
		1/5 -	1/10 -	1/5 +	1/10 +	1/5 -	1/10 -	1/5 +	1/10 +	1/5 -	1/10 -	1/5 +	1/10 +	1/5 -	1/10 -	1/5 +	1/10 +	1/5 -	1/10 -	1/5 +	1/10 +	1/5 -	1/10 -	1/5 +	1/10 +
30 °C	mv	2.71	5.35	6.91	5.49	2.92	3.79	7.09	9.19	1.30	3.09	6.37	8.03	1.56	1.78	2.87	3.22	1.41	1.33	2.37	4.62	0.82	0.93	2.20	4.69
	sd	0.40	1.08	0.39	0.53	0.29	0.36	1.01	0.33	0.05	0.36	0.87	0.37	0.27	0.30	0.65	0.38	0.13	0.03	0.12	0.60	0.02	0.10	0.14	0.54
25 °C	mv	1.44	1.83	1.65	1.00	3.86	6.40	10.44	10.07	3.05	6.14	10.18	10.67	1.31	0.77	0.49	0.31	1.71	2.89	4.03	6.08	1.37	1.77	3.75	6.17
	sd	0.01	0.04	0.03	0.06	0.05	0.19	0.11	0.41	0.20	0.23	0.40	0.66	0.04	0.02	0.03	0.02	0.24	0.20	0.29	0.39	0.08	0.09	0.13	0.51
20 °C	mv	1.92	1.77	1.09	0.88	8.98	9.39	9.31	9.82	6.50	13.06	11.35	10.75	1.28	1.14	0.40	0.28	4.06	6.43	8.32	10.70	2.71	4.85	10.10	13.90
	sd	0.28	0.32	0.03	0.08	0.61	0.33	0.74	1.34	0.72	1.26	0.24	0.14	0.04	0.07	0.06	0.04	0.51	0.67	0.43	1.80	0.17	0.84	1.74	1.12

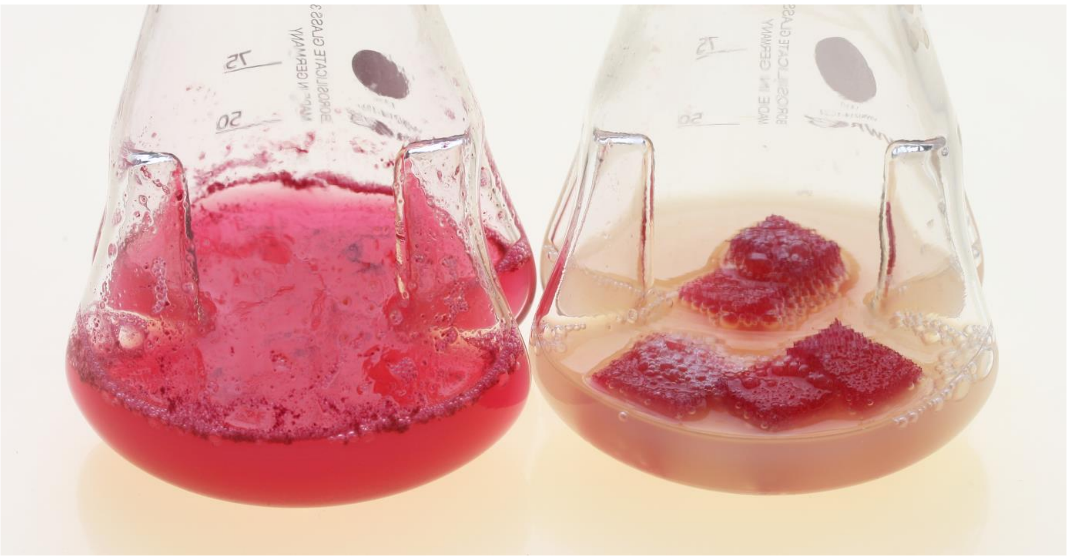

Supplementary Figure S5 | Adsorption of prodigiosin by polyurethane foam in culture broth.

For visualization of prodigiosin adsorption by polyurethane foam in culture broth, *P putida* pig-r2 was cultivated with and without polyurethane foam cubes in LB medium (shaking at 30 °C for 24 h). As LB medium is lighter in color than TB, it was chosen to enable higher contrast. Furthermore, the culture broth was acidified with HCl directly prior of taking the picture for better visualization of prodigiosin. Prodigiosin is adsorbed by the polyurethane foam cubes and can easily be harvested for further purification.

Supplementary Table S2 | Comparison of prodigiosin extraction procedures with and without polyurethane foam cubes as *in situ* adsorbent.

Characteristics of crude extracts are given, referring to the obtained products after prodigiosin extraction from PU or cells and subsequent two-phase extraction, following the here applied protocols (see methods section). * Prodigiosin titers in cultures with PU can only be estimated based on the amount remaining in the cell fraction after removal of PU (determined to be < 1 mg/L), and recovered prodigiosin from PU. After loading of PU into the Soxhlet extractor, full recovery can only be assumed since purification of the bright red compound can be followed visually. Therefore, the recovery can likewise only be estimated. ♦ *Hands-on* working time refers to the procedures as conducted with common laboratory material by one person. Specifically, the PU-based workup consists of following steps: PU isolation by sieving, PU wringing, Soxhlet-extraction, two-phase extraction and solvent evaporation. In case of the procedure without PU, the workup consists of: Cell harvesting, cell pellet extraction, two-phase extraction and solvent evaporation. *Hands-off* times in procedures like automated Soxhlet extraction or rotary solvent evaporation which do not require attendance are not included.

	**with PU**	**without PU**
**Prodigiosin titer in culture**	~ 48 mg/L*	24 ± 8 mg/L
**Prodigiosin yield in crude extract**	47 ± 6 mg/L	23 ± 9 mg/L
**Recovery of prodigiosin from culture to crude extract**	> 97%*	ca. 94%
**Purity of crude extract**	37 ± 1%	9 ± 2%
**Special costs per 500 mL batch**	PU: 0.14 €	high-g centrifuge tubes: 1.20 €
***Hands-on* working time per 500 mL batch♦**	ca. 45 min	ca. 95 min

IV Analytical comparison of biotechnologically produced prodigiosin to the chemical reference

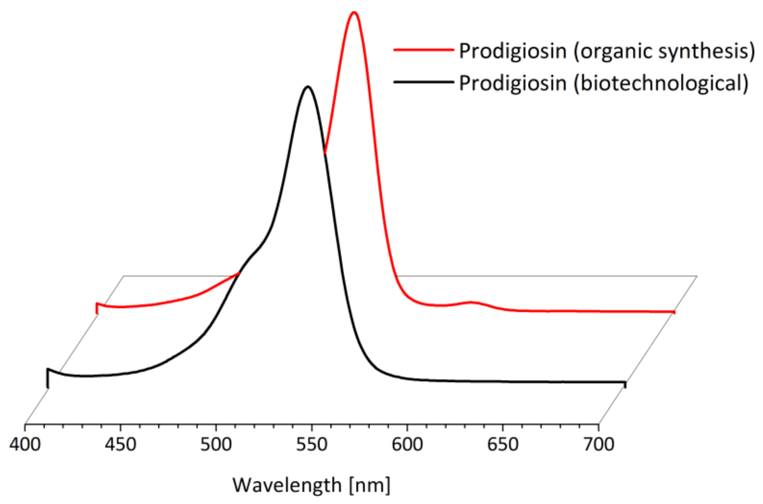

Supplementary Figure S6 | UV/VIS spectrum of chemical and biotechnological (crude extract). prodigiosin. The absorption was measured in acidified ethanol (4% v/v of 1 N HCl) (λ_{max} = 535 nm for both samples).

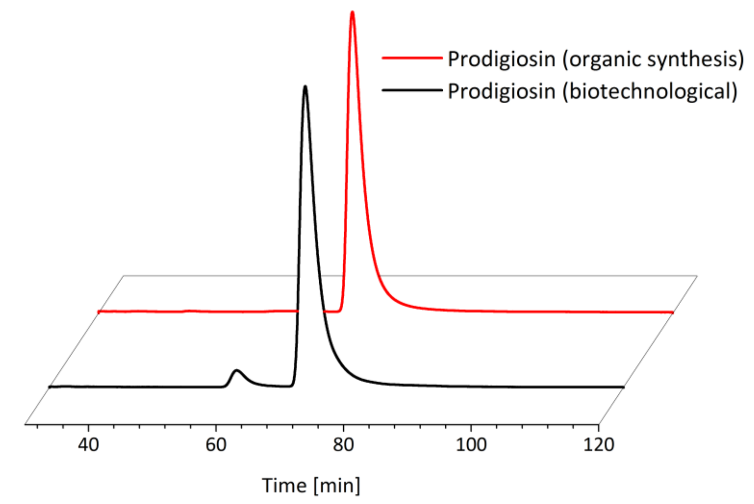

Supplementary Figure S7 | HPLC chromatogram of chemical and biotechnological (crude extract). prodigiosin. Column: Chiralpak® IA (250 x 4.6 mm, 5 µm; Flow: 0.5 mL min^{-1}; Detection: 465 nm; Solvent: Heptane and 2-propanol (80:20). t_R = 69.95 min (for both samples).

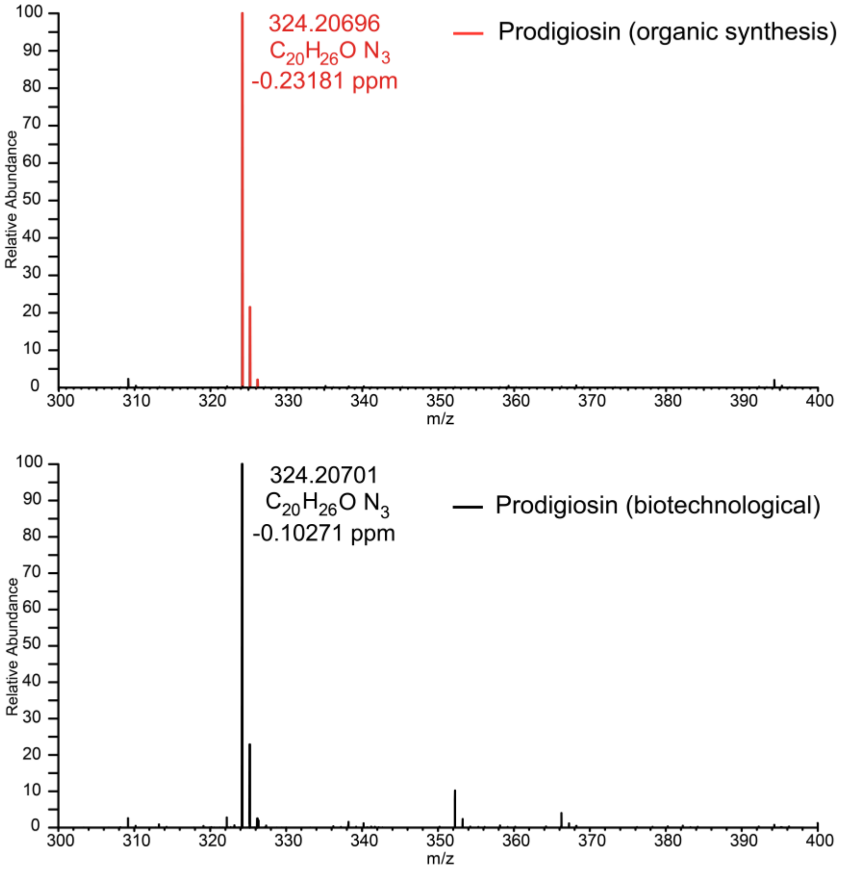

Supplementary Figure S8 | HRMS spectra of chemical and biotechnological (crude extract) prodigiosin.

Supplementary References

Aldrich, L.N., Dawson, E.S., and Lindsley, C.W. (2010). Evaluation of the Biosynthetic Proposal for the Synthesis of Marineosins A and B. *Organic Letters* 12, 1048-1051. doi:10.1021/ol100034p

Beauchemin, A.M., Moran, J., Lebrun, M.E., Seguin, C., Dimitrijevic, E., Zhang, L., and Gorelsky, S.I. (2008). Intermolecular cope-type hydroamination of alkenes and alkynes. *Angewandte Chemie-International Edition* 47, 1410-1413. doi:10.1002/anie.200703495

Dairi, K., Tripathy, S., Attardo, G., and Lavallée, J.-F. (2006). Two-step synthesis of the bipyrrole precursor of prodigiosins. *Tetrahedron Letters* 47, 2605–2606. doi:10.1016/j.tetlet.2006.02.035.

Durchschein, K., Ferreira-da Silva, B., Wallner, S., Macheroux, P., Kroutil, W., Glueck, S. M., and Faber, K. (2010). The flavoprotein-catalyzed reduction of aliphatic nitro-compounds represents a biocatalytic equivalent to the Nef-reaction. *Green Chemistry* 12, 616–619. doi:10.1039/B922691E.

Hajipour, A. R., Mallakpour, S. E., and Imanzadeh, G. (1999). A Rapid and Convenient Synthesis of Oximes in Dry Media under Microwave Irradiation. *Journal of Chemical Research, Synopses*, 228–229. doi:10.1039/A806359A.

Haynes, S. W., Sydor, P. K., Stanley, A. E., Song, L., and Challis, G. L. (2008). Role and substrate specificity of the *Streptomyces coelicolor* RedH enzyme in undecylprodiginine biosynthesis. *Chemical Communications* 28, 1865–1867. doi:10.1039/B801677A.

Trofimov, B. A., Mikhaleva, A. I., Vasil'ev, A. N., Korostova, S. E., and Shevchenko, S. G. (1985). Pyrroles from ketoximes and acetylene. 29. Synthesis of alkylpyrroles from dialkylketoximes and dichloroethane by reaction with KOH-DMSO. *Chemistry of Heterocyclic Compounds* 21, 46–49. doi:10.1007/BF00505898.

Williamson, N.R., Simonsen, H.T., Ahmed, R.a.A., Goldet, G., Slater, H., Woodley, L., Leeper, F.J., and Salmond, G.P.C. (2005). Biosynthesis of the red antibiotic, prodigiosin, in *Serratia*: identification of a novel 2-methyl-3-n-amyl-pyrrole (MAP) assembly pathway, definition of the terminal condensing enzyme, and implications for undecylprodigiosin biosynthesis in *Streptomyces*. *Molecular Microbiology* 56, 971-989. doi:10.1111/j.1365-2958.2005.04602.x

Yu, C., Jiao, L., Tan, X., Wang, J., Xu, Y., Wu, Y., Yang, G., Wang, Z., and Hao, E. (2012). Straightforward Acid-Catalyzed Synthesis of Pyrrolyldipyrromethenes. *Angewandte Chemie International Edition* 51, 7688–7691. doi:10.1002/anie.201202850.
